# Feasibility and user evaluation of HopeBot: An LLM-powered conversational chatbot for depression screening

**DOI:** 10.1371/journal.pdig.0001446

**Published:** 2026-06-25

**Authors:** Zhijun Guo, Alvina Lai, Julia Ive, Alexandru Petcu, Yutong Wang, Luyuan Qi, Johan H. Thygesen, Kezhi Li

**Affiliations:** 1 Institute of Health Informatics University College, London, London, United Kingdom; 2 Lancashire & South Cumbria NHS Foundation Trust, Bamber Bridge, ‌United Kingdom; 3 University College London Hospital NHS Foundation Trust, London, United Kingdom; Massachusetts General Hospital, UNITED STATES OF AMERICA

## Abstract

Clinician-administered Patient Health Questionnaire-9 (PHQ-9) interviews allow clarification of ambiguous responses but are resource-intensive and difficult to scale for routine use. Self-administered versions are widely adopted for depression screening yet offer little opportunity for interaction or clarification, which may limit engagement and scoring accuracy. We developed HopeBot, a conversational chatbot powered by a large language model (LLM) that delivers the PHQ-9 via text or voice, providing real-time clarification and safety guidance through a retrieval-augmented generation (RAG) layer drawing on validated psychological and helpline resources. The system aims to extend access to structured screening rather than replace clinician judgment. In a within-subject feasibility study, 132 adults from two countries completed both self-administered and chatbot-assisted PHQ-9 assessments followed by a 25-item evaluation survey. Chatbot and self-report scores showed high concordance (intraclass correlation coefficient = 0.92; median absolute difference = 1 point), indicating faithful replication of scoring. Of these participants, 75 completed a comparative feedback module (most with identical scores were not prompted for comparison); 71% (n = 53) reported greater confidence in chatbot-assisted scores, citing clearer structure, interpretive guidance, and a supportive tone. Mean usability ratings (0–10) were 8.4 for comfort, 7.7 for voice clarity, 7.6 for handling sensitive topics, and 7.4 for recommendation helpfulness; the latter varied significantly by employment status and prior experience with mental-health services. The self-administered PHQ-9 served as a pragmatic comparator reflecting real-world digital screening, allowing evaluation of whether the chatbot could faithfully reproduce the questionnaire’s structure and scoring accuracy rather than diagnostic validity. These findings indicate that an LLM-powered conversational agent with RAG-based grounding can feasibly and acceptably administer the PHQ-9 with strong score concordance relative to self-report, suggesting potential as a scalable adjunct for early, low-burden depression screening. Further validation against clinician-administered assessments in real-world workflows is warranted.

Trial registration

ClinicalTrials.gov Identifier NCT06801925

## Author summary

Depression affects millions of people worldwide, yet early detection remains low. Most screening approaches rely on self-administered questionnaires such as the Patient Health Questionnaire-9, which, while convenient, offer little opportunity for clarification or emotional expression. This lack of interactivity can make screening feel impersonal and reduce engagement. We developed HopeBot, an interactive chatbot that makes depression screening more conversational and supportive. Instead of asking users to tick boxes, HopeBot guides them through the same nine questions in everyday language. When answers are unclear, it asks gentle follow-up questions, and when signs of distress appear, it provides supportive feedback and professional helpline information. The aim is not to replace clinicians but to make screening more personal and accessible for those who might otherwise avoid it. In a study of 132 adults from the United Kingdom and China, HopeBot showed strong consistency with self-reported scores from traditional questionnaires while providing a more structured and engaging experience. Many participants reported greater confidence in the results generated by HopeBot, noting its clearer structure and more empathic tone. Conversational systems like HopeBot could potentially make early depression screening more accessible and supportive, particularly in settings with limited professional resources.

## Introduction

Depression is a major global health issue characterised by persistent low mood, loss of interest or pleasure in daily activities, and impaired cognitive and emotional functioning [1]. It often results in sleep disturbances, fatigue, social withdrawal, and reduced occupational or academic productivity, imposing significant emotional and economic burdens on individuals and society [2]. The World Health Organisation (WHO) estimates that depression affects approximately 3.8% of the global population [1]. Recent burden-of-disease data indicate comparable 12-month prevalence in the United Kingdom (UK) (around 4–5%) [3] and slightly lower rates in China (approximately 3–4%) [4]. Broader national surveys that assess symptoms rather than clinical diagnoses report higher figures—up to one in five adults in the UK experience depressive or anxiety symptoms annually [5]. Yet only about half receive minimally adequate counselling or antidepressant treatment [6]. Delayed identification of depression can exacerbate symptoms, increasing risks for chronic disability and suicide, with over 700,000 individuals dying by suicide annually due to depression [1]. This underscores the critical importance of timely screening and intervention. Traditional approaches such as psychological counselling and psychiatric assessments typically require trained professionals, extensive time commitments, and substantial financial resources [7,8], posing notable barriers in resource-limited settings and economically disadvantaged populations [9,10]. Additionally, societal stigma associated with mental illness frequently discourages affected individuals from actively seeking care, further impeding timely identification and treatment [7].

The Patient Health Questionnaire-9 (PHQ-9) is one of the most widely used and validated instruments for screening and grading depressive symptoms. Meta-analytic evidence indicates that at the conventional cut-off score of 10, the sensitivity of the PHQ-9 ranges from 77% to 88% and specificity from 85% to 94%, depending on the population and administration context [11,12]. While the instrument demonstrates robust psychometric performance across diverse demographics, its accuracy and clinical utility are highly influenced by the mode of administration [13,14]. Clinician-administered and semi-structured formats facilitate clarification of ambiguous responses and the detection of suicidality or comorbid psychiatric features, whereas self-administered formats rely exclusively on respondent interpretation without adaptive guidance [15]. This reliance may introduce inconsistency in responses, particularly in individuals with lower health literacy or fluctuating emotional states. Furthermore, static delivery formats, whether paper-based or digital, offer limited interactivity and cannot tailor the presentation of items to user needs in real time [16,17]. These limitations underscore a need for novel administration modalities that preserve the diagnostic integrity of the PHQ-9 while enhancing procedural efficiency, standardisation of delivery, user engagement, and scalability across diverse populations.

Conversational agents powered by large language models (LLMs)—neural network–based systems designed to generate and interpret natural language—have emerged as a promising means of addressing limitations in traditional mental health screening [18]. Trained on extensive corpora, LLMs can generate contextually appropriate and syntactically coherent responses, support real-time clarification of user input [19,20], adapt to individual linguistic patterns, and maintain coherence over extended interactions [21,22]. These capabilities are particularly valuable in mental health contexts, where communication is often ambiguous, incomplete, or emotionally nuanced [23,24].

The use of LLMs in mental health assessment and support, however, raises important concerns. These include the risk of inaccurate or unsafe outputs, opaque reasoning processes, and lack of real-time oversight in high-risk situations such as suicidal disclosures [22,25]. Additional ethical challenges include data privacy, informed consent, and the interpretability of model-generated recommendations [22]. These limitations highlight the need for rigorous evaluation and transparent design to ensure safe and ethical use of LLMs in mental health contexts.

Several prior chatbot-based depression screening systems, such as DEPRA [21], IGOR [26], Perla [18], Marcus [27], and EmoScan [28] have demonstrated initial feasibility using structured frameworks and standardised assessments (e.g., PHQ-9, SIGH-D, IDS-C). DEPRA and IGOR both rely on predefined conversational intents to ensure procedural safety, yet this rule-based design limits conversational flexibility and restricts users from elaborating on ambiguous emotional states [21,26]. Perla similarly integrates the PHQ-9 within a fixed intent architecture that constrains adaptation to user language variability [18], while Marcus, though employing BERT-based classifiers, still lacks transparent item-level scoring and the ability to manage uncertain or incomplete user responses [27]. EmoScan aims to improve linguistic generalisability through synthetic clinical dialogues, but it does not directly incorporate standardised diagnostic tools such as the PHQ-9 [28]. Collectively, these systems confirm the feasibility of chatbot-mediated screening yet remain limited in their ability to deliver psychometrically faithful, context-aware PHQ-9 administration with transparent reasoning and dynamic clarification.

To address these methodological gaps, we developed HopeBot, an LLM (GPT-4o)-powered conversational system that administers the PHQ-9 within a flexible, empathic dialogue while preserving the validated structure and scoring logic of the original instrument. HopeBot integrates retrieval-augmented generation (RAG)—a framework in which model responses are grounded by retrieving relevant information from external knowledge sources—with structured prompt design to support flexible, empathic dialogue while preserving the validated structure and scoring logic of the PHQ-9 [7]. By grounding clarifications and feedback in authoritative psychological and helpline sources, this design aims to improve the reliability and interpretability of chatbot responses and to mitigate potential hallucinations, defined as the generation of plausible but factually unsupported outputs [29,30]. This evidence-grounded design mitigates the inconsistencies and unverifiable outputs often observed in typical open-ended ChatGPT interactions [31]. Unlike prior rule-based or intent-driven chatbots, HopeBot interprets free-text responses adaptively, issues real-time clarifications when inputs are ambiguous or incomplete, and explicitly maps user responses to PHQ-9 categories (A–D) and corresponding scores. This format-aligned administration design, defined as a methodological framework that preserves the validated structure, sequence, and scoring logic of a standardised psychological instrument rather than reformulating assessment items into unconstrained conversational prompts, while enabling natural conversational interaction, maintains scoring fidelity while improving procedural efficiency and interpretability. In addition, HopeBot supports brief rapport-building dialogue before and after formal assessment, facilitating a natural conversational transition without altering the standard PHQ-9 protocol or its clinical interpretation framework.

We conducted a prospective mixed-methods study with 132 participants from diverse educational and cultural backgrounds from two countries (UK and China) to evaluate the feasibility and score-level concordance between HopeBot-assisted and self-administered PHQ-9 assessments. Because both formats used the same validated instrument, the study focused on screening-level concordance rather than diagnostic validity; establishing diagnostic accuracy would require comparison with a clinician-administered reference. The present work, therefore, represents a format-level validation framework, positioned as a methodological bridge between conventional self-assessment and clinician-guided evaluation. Beyond quantitative concordance, user perceptions of clarity, comfort, and trust were explored to assess the acceptability and usability of LLM-assisted screening as a complementary—not replacement—tool for early depression detection.

## Methodology

### Ethics statement

This study was reviewed and approved by the University College London (UCL) Research Ethics Committee following submission of a high-risk application (ID: 26133.001). An amendment and extension to the original protocol was subsequently approved, with ethics coverage extended until 29 January 2026. All procedures were conducted in accordance with institutional ethical standards and the principles outlined in the Declaration of Helsinki [32]. Prior to participation, informed consent was obtained from all individuals. The study was also prospectively registered on ClinicalTrials.gov under reference number NCT06801925.

### Study design and reporting standards

This feasibility and format-level concordance study was reported with reference to the GRRAS guidelines (Guidelines for Reporting Reliability and Agreement Studies) [33], which are appropriate for assessing agreement between measurement formats (see S1 Table). A small number of transparency-related elements from the STARD framework (Standards for Reporting Diagnostic Accuracy Studies) [34] were consulted solely to support clear documentation of study procedures and data flow (see S2 Table). The self-administered PHQ-9 was treated as a pragmatic comparator for evaluating scoring fidelity rather than as a clinical reference standard. HopeBot was evaluated as a supplementary screening modality to examine feasibility, usability, and consistency with the validated PHQ-9 instrument, not as a diagnostic tool.

### Chatbot system design

HopeBot served as the index test and was designed to administer the PHQ-9 through a dynamic, LLM-driven conversational interface while preserving full alignment with the validated scoring structure of the reference standard. It was evaluated solely for diagnostic concordance and feasibility and was not intended to support clinical decision-making or treatment recommendations. Unlike previous rule-based chatbot systems that follow predefined conversational intents, HopeBot integrates an RAG architecture to enable open-ended dialogue that remains grounded in validated psychological knowledge sources [35]. This knowledge-grounded design enhances interpretability, reduces hallucination risk, and enables adaptive clarification in response to user uncertainty [30].

**Fig 1:**
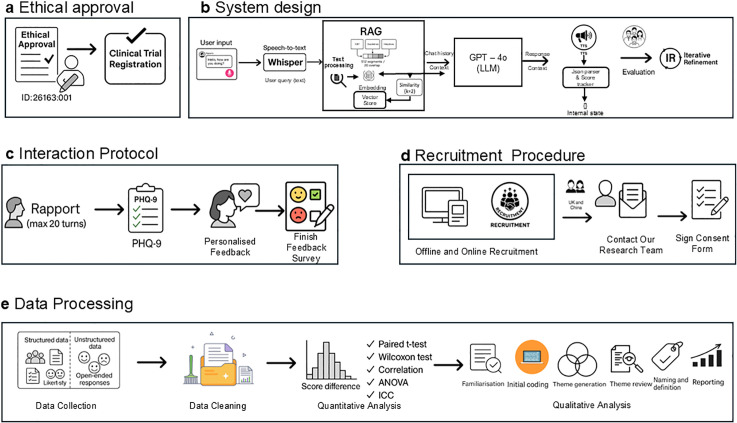
Overview of the HopeBot study workflow, from ethical approval to data analysis. The figure summarises the end-to-end design of a format-aligned, conversational PHQ-9 screening system. a, Ethical approval and clinical trial registration. b, System design of HopeBot. The RAG-based conversational pipeline transcribes user speech via Whisper and retrieves CBT-informed context from a ChromaDB vector store (top k = 2). GPT-4o integrates user input, retrieved context, and prior dialogue history to generate responses. The system produces parallel outputs: natural language responses are synthesised via TTS-1, while structured JSON outputs are parsed to enable real-time PHQ-9 score tracking. All processes were coordinated asynchronously and refined through expert evaluations. c, Interaction protocol. User conversations followed a three-phase structure: rapport building (max 20 turns), PHQ-9 administration, and personalised feedback, followed by a feedback survey. d, Recruitment procedure. Participants aged 18–70 were recruited via online and offline strategies. e, Data processing and analysis. Structured (e.g., Likert-scale) and unstructured (e.g., open-ended) data were cleaned and analysed. Qualitative analysis followed Braun and Clarke’s [36] thematic analysis framework.

**Fig 2:**
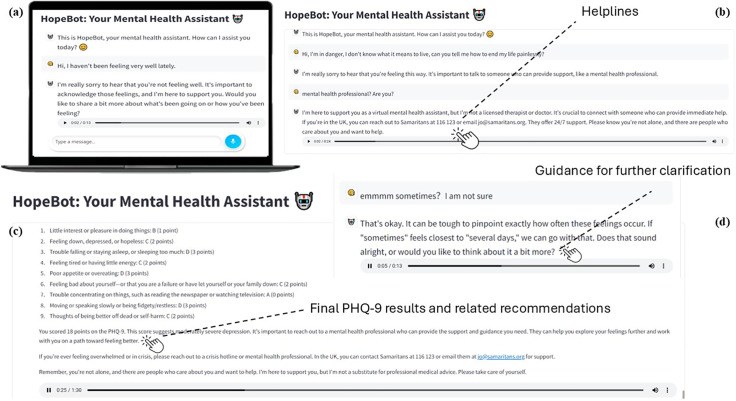
HopeBot interface and representative outputs. a, The interface allows users to engage with the chatbot through either typed or spoken input. During system response, text is rendered incrementally in a character-by-character fashion, followed by automatic audio playback via OpenAI’s TTS-1 (voice: ‘sage’). Playback begins after the full transcript is displayed and can be paused or interrupted by the user at any time. To reduce cognitive load and ensure user privacy, particularly in the context of sensitive mental health conversations, only the most recent audio response was accessible during each turn. Previous responses were neither stored nor replayable. b, Safety handling triggered by crisis-related language, in which HopeBot provides supportive responses and redirects users to appropriate mental health helplines. c, Final PHQ-9 output with item-level scores, total severity classification, and tailored support recommendations, presented in both audio and text. d, Clarification prompt issued when user responses are ambiguous, illustrating how HopeBot seeks additional information to support accurate PHQ-9 scoring.

The complete system workflow is illustrated in Fig 1. The user interface was developed using Streamlit [37] to enable synchronous multimodal input via keyboard or microphone (Fig 2). Voice input was processed using OpenAI’s Whisper [38] automatic speech recognition model, and system responses were synthesised into audio using the OpenAI TTS-1 text-to-speech system [39]. All components of transcription, generation, and rendering were managed within an asynchronous event loop to preserve natural turn-taking and maintain interactional fluidity.

The system supported both English and Mandarin through GPT-4o’s native multilingual capabilities. Responses were generated directly in the input language without translation. Mandarin outputs were produced from Chinese prompts, and audio synthesis was handled by a general-purpose text-to-speech engine. All interactions were generated using the OpenAI GPT-4o model accessed via the OpenAI API (model identifier: “gpt-4o”) [40]. Data collection and system evaluation were conducted within a fixed study window (March–April 2025), during which the same model endpoint and prompt configuration were used throughout. To minimise the impact of model updates, the system prompt was fixed prior to data collection, and no adaptive prompt or model changes were introduced during the study period. While response stochasticity is inherent to LLMs, system behaviour was monitored during internal testing through repeated end-to-end trial runs and manual review of conversation logs to verify consistent task sequencing, PHQ-9 item coverage, and scoring logic. No substantive deviations in task flow or score computation were observed during the study period.

To ground the chatbot’s responses in validated psychological knowledge, we implemented a multi-source RAG layer using LangChain and Chroma [41]. Four primary data sources were assembled: (i) A curated corpus of 34 anonymised Cognitive Behavioural Therapy (CBT) session transcripts compiled from publicly available training materials, including YouTube-based simulations, therapist role-plays, and anonymised transcripts from online repositories. (ii) The full text of A Therapist’s Guide to Brief CBT was included to ensure coverage of structured, evidence-based strategies [42]. (iii) Two public corpora were integrated to support emotional relevance: ESConv, an English dialogue dataset annotated for user emotions and support strategies [43]; and PsyQA_example, a Chinese mental health QA corpus covering topics such as depression and anxiety [44]. (iv) Bilingual helpline directories from the UK and China, containing validated contact information and service descriptions.

For retrieval, these sources were consolidated into three Chroma vector stores according to functional role: a CBT-oriented store integrating CBT transcripts and guideline-based materials; an emotional-support store integrating ESConv and PsyQA_example; and a helpline resource store integrating UK and China helpline directories. The CBT vector store integrated publicly accessible materials selected for their structured, clinically grounded nature [45,46], including annotated scripts (e.g., from learn.problemgambling.ca), case dialogues, and educational videos by licensed clinicians. Subtitles from video content were extracted, speaker-segmented, and cleaned. All resources were used solely for research in accordance with their stated terms and screened for alignment with core CBT principles such as socratic questioning, cognitive restructuring, and behavioural activation [45,46]. Retrieved evidence was used internally to guide response generation but was not displayed to users in real time. Instead, HopeBot synthesised the retrieved content into coherent natural language responses designed to remain consistent with the supporting sources. This design helped ensure that outputs were grounded in authoritative psychological materials while maintaining conversational fluency and reducing users’ cognitive load.

All documents were pre-processed and indexed during offline corpus construction using a recursive character-level chunking strategy with 512-token segments and a 20% overlap. Text embeddings, used for semantic retrieval, were generated using the text-embedding-3-small model [47]. At each conversational turn, the user’s most recent utterance was used as the retrieval query. Retrieval was performed across three Chroma vector stores corresponding to the curated CBT corpus, public emotional-support datasets, and helpline resources. For each store, the top-k most relevant passages were retrieved (k = 2 per store; up to six passages per turn). No additional similarity thresholding or reranking was applied.

The retrieved passages were concatenated in newline-separated form and injected into the system prompt as contextual grounding, alongside the full conversational history. Response generation was governed by a structured, task-oriented system prompt that explicitly controlled dialogue flow, PHQ-9 item administration, response classification, scoring logic, and safety constraints. The augmented prompt was then passed to GPT-4o to generate responses. The system prompt was iteratively refined during expert review and fixed prior to deployment; the final version is provided in S1 Text. This architecture enabled the chatbot to alternate seamlessly between open-ended therapeutic dialogue and structured screening procedures, while maintaining psychological validity and factual coherence.

HopeBot followed a predefined three-phase protocol: (1) rapport building (maximum 20 turns), (2) PHQ-9 administration, and (3) personalised feedback. PHQ-9 items were delivered sequentially in accordance with the validated instrument structure. GPT-4o was used solely to interpret user responses and assign them to one of four PHQ-9 response categories (A–D), while numerical scoring (0–3 per item) and total score aggregation were executed deterministically by a backend function independent of the language model. This ensured fully reproducible scoring and eliminated hallucination-related arithmetic errors. When user input was ambiguous, the model generated clarification prompts before classification. Once all items were completed, the backend automatically calculated the total score, and GPT-4o generated the final interpretative summary, including item-level responses, severity classification based on validated PHQ-9 thresholds, and tailored recommendations. The entire process was fully automated, with no human involvement in scoring or feedback generation. On average, GPT-4o generated each conversational response in 1.47 ± 0.30 seconds, and speech synthesis required an additional 2.36 ± 0.49 seconds, resulting in a total latency of approximately 3.83 seconds per interaction turn.

The prototype was reviewed by four domain experts, including a practising NHS clinical psychiatrist in the UK, two doctoral researchers at UCL, and a licensed mental health counsellor in China. Reviewers noted that the system maintained acceptable response latency and did not disrupt conversational flow. Their feedback also addressed scoring validity, linguistic tone, empathy, and the handling of ambiguous responses, informing iterative refinements before participant deployment.

To ensure ethical and secure operation, HopeBot incorporated safeguards addressing emotional safety, crisis response, and data privacy throughout all interactions. All conversations were processed within a secure, institution-approved research environment, and no identifiable personal health data were stored, retained for clinical purposes, or transmitted beyond the research server. The system operated in compliance with the UK GDPR research-use provisions (Article 89 of the EU GDPR), as covered by the study’s ethics approval. In practice, all data were pseudonymised, stored securely within the institutional research environment, and processed solely for non-clinical research purposes. Additional details on safety protocols, crisis handling, and data flow architecture are provided in S2 Text.

### Evaluation: Participant Recruitment and Procedure

Participant recruitment was carried out concurrently in the UK and China using online and offline strategies to ensure demographic diversity. Eligible participants were adults aged 18–70 years who provided informed consent and were able to complete the study in either English or Mandarin. Advertisements were disseminated via social media platforms (e.g., Facebook, X, Xiaohongshu) and printed posters at universities and community venues. Interested individuals received a participant information sheet and consent form before enrolment.

Both the comparator and index test were completed during a single study session to minimise temporal variation in depressive symptom reporting. Participants first completed the self-administered PHQ-9 using the validated English or Mandarin version, which served as the reference framework for subsequent comparison with the HopeBot-assisted administration. They were not exposed to HopeBot content or scoring logic prior to this step, ensuring unbiased self-reporting. Following completion of the reference standard within the same session, participants proceeded to the HopeBot-assisted PHQ-9 using either typed or spoken input on a desktop or mobile device. The chatbot interaction followed the predefined three-phase protocol (rapport building, PHQ-9 delivery, personalised feedback) and lasted approximately 25 minutes. After the HopeBot session, participants completed a structured post-interaction survey comprising five demographic items, two PHQ-9 score entries (self-administered and HopeBot-assisted), and eighteen Likert-scale and open-ended questions assessing clarity, comfort, trust, empathy, and perceived usefulness (see S3 Text). Participants were encouraged to elaborate on their responses. Survey completion took on average 35 minutes. Data were collected between 1 March and 3 April 2025.

A total of 191 individuals were initially enrolled. Submissions were excluded if they (i) completed less than 80% of the questionnaire (n = 32), (ii) submitted incoherent or AI-generated responses (n = 12), or (iii) provided non-substantive answers to open-ended questions, such as single-word replies, vague affirmations (e.g., “good” or “helpful”), or content copied from external sources (n = 15). After quality screening, 132 responses were retained for analysis. In the final analytic sample, all PHQ-9 items were completed, and no item-level missing data were present in either the self-administered or HopeBot-assisted assessments. For Likert-scale user evaluation items (e.g., Q17–Q20), non-numeric responses such as “I don’t know” were retained in the dataset but excluded from item-level quantitative analyses; summary statistics and tests were computed using available numeric responses only, without imputation.

### Data analysis method

Descriptive statistics were generated for all structured survey responses using Python 3.11. To evaluate the consistency between self-administered and HopeBot-assisted PHQ-9 scores, a within-subject design was adopted, treating the validated self-administered PHQ-9 as the reference format for comparison. Both absolute and signed score differences were calculated, and measures of central tendency (mean, median) and dispersion (standard deviation [SD], interquartile range [IQR]) were reported [48]. Paired t-test and Wilcoxon signed-rank tests were conducted to compare PHQ-9 scores between formats [49]. Agreement between the two modes of administration was quantified using Spearman’s rank correlation coefficient [50] and the intraclass correlation coefficient ICC(3,1) (two-way mixed effects, absolute agreement), a measure of agreement between repeated measurements within individuals [51].

Exploratory subgroup analyses were pre-specified for recruitment countries (UK vs China) and baseline PHQ-9 severity (<10 vs ≥ 10). These two moderators were selected a priori because they are most relevant both theoretically (reflecting linguistic and psychometric variability) and operationally (influencing cross-cultural deployment and implementation contexts). Between-group differences in absolute score difference were tested using two-sided Mann–Whitney U tests [52] with Holm–Bonferroni correction for multiple comparisons (α = 0.05) [53]. For non-parametric comparisons, effect size (r) was reported to quantify the magnitude of between-group differences beyond statistical significance and interpreted using conventional thresholds (0.1 small, 0.3 medium, 0.5 large) [54]. Additional exploratory analyses of other demographic variables are provided in the S3 Table.

To explore associations between demographic factors and user ratings across four key outcomes (Q17–Q20), independent samples t-tests and one-way ANOVA were applied, depending on the variable structure [55]. All significance tests were two-sided with an α threshold of 0.05. Effect sizes were reported as Cohen’s d [56] for t tests and Eta Squared (η²) [57] for ANOVA to quantify the magnitude of associations. Multilevel demographic variables were dichotomised a priori to maintain expected cell counts ≥ 5 (e.g., recruitment country UK vs China; age ≤ 34 vs ≥ 35 years; ethnicity White vs non-White; education degree vs non-degree). Each demographic factor was cross-tabulated (2 × 2) against three binary endpoints: (i) perceived trustworthiness of PHQ-9 scores, (ii) preferred screening modality, and (iii) intention to recommend or reuse HopeBot. Pearson’s χ² test with Yates’ correction was used when appropriate [58]; otherwise, Fisher’s exact test was applied [59,60].

Open-ended responses were thematically analysed using Braun and Clarke’s six-phase framework [36] (see Fig 1). Coding was conducted inductively by the first author to allow themes to emerge from the data. To ensure analytic rigour, a second qualitative researcher (KL) independently reviewed the codes. Inter-coder agreement was 86%, indicating good consistency. Discrepancies in code assignment or theme mapping were resolved through iterative discussion between coders, involving joint review of the original responses, clarification of code definitions within the codebook, and refinement of theme boundaries until consensus was reached. A full codebook outlining code definitions, inclusion criteria, and exemplar quotes is provided in S4 Text. Word frequency statistics were computed using Python to support theme validation and lexical salience analysis; the distribution of word frequencies is presented in S4 Table and S5 Table.

## Results

### Participant characteristics

Of the 132 participants included in the final analysis, 68 (51.5%) were recruited in the UK. 75% were under 45 years of age, 54.5% identified as female, and 56.1% as Asian or Asian British, while 38.6% identified as White. Most participants held an undergraduate or postgraduate degree (88.7%) and were either in full-time employment (59.1%) or full-time education (22.7%). Familiarity with LLMs was high overall, with 85 participants (64.4%) describing themselves as regular users, and only 2 (1.5%) reporting no prior experience. In total, 56 participants (42.4%) had previously interacted with chatbot technologies, most (48/56, 85.7%) reported using general-purpose LLMs (e.g., ChatGPT, Doubao) for emotional disclosure or mental health–related interactions, rather than specialised mental health chatbots. Prior experience with conventional mental health support was reported by 26 participants (19.7%). The majority of participants scored in the minimal-to-mild range on both self-administered (70.4%) and HopeBot-assisted PHQ-9 assessments (72%) (see Table 1).

**Table 1:** Sociodemographic and background characteristics of the survey respondents (n = 132).

Characteristic	Category	n	%
**Country of Recruitment**	UK	68	51.5
	China	64	48.5
**Age group (years)**	18 – 24	27	20.5
	25 – 34	40	30.3
	35 – 44	32	24.2
	45 – 54	19	14.4
	55 – 64	12	9.1
	65 – 70	2	1.5
**Gender**	Female	72	54.5
	Male	60	45.5
**Ethnicity**	Asian or Asian British	74	56.1
	White	51	38.6
	Black / Black British / Caribbean	4	3.0
	Mixed / Multiple groups	2	1.5
	Prefer not to say	1	0.8
**Highest education**	Undergraduate degree	81	61.4
	Post-graduate degree (Master’s/PhD)	36	27.3
	Further education (e.g., A-levels/NVQ)	13	9.9
	No formal qualification / Prefer not to say	2	1.5
**Employment status**	Full-time employment	78	59.1
	Full-time education/training	30	22.7
	Part-time employment	12	9.1
	Looking after home	5	3.8
	Other / Retired	6	4.5
	Prefer not to say	1	0.8
Familiarity with LLMs*	Regular user	85	64.4
	Heard of / tried once	29	22.0
	Occasional user	8	6.1
	Technical expert	8	6.1
	No experience	2	1.5
**Mental health chatbot experience**	Yes	56	42.4
	No	76	57.6
**Previous mental health support experience**	Yes	26	19.7
	No	106	80.3
**PHQ-9 Severity Result – Self-report**	Minimal/None (0–4)	51	38.6
	Mild (5–9)	42	31.8
	Moderate (10–14)	25	18.9
	Moderately Severe (15–19)	9	6.8
	Severe (20–27)	5	3.8
**PHQ-9 Severity Result – HopeBot**	Minimal/None (0–4)	48	36.4
	Mild (5–9)	47	35.6
	Moderate (10–14)	24	18.2
	Moderately Severe (15–19)	9	6.8
	Severe (20–27)	4	3.0

Data are presented as counts and percentages (n, %), summarising participant demographics, background characteristics, and baseline PHQ-9 severity distributions. *LLM = large language model.

### System performance and RAG-driven interaction characteristics

While administering the PHQ-9, HopeBot actively sought clarification when user input was vague or non-categorical. For example, responses such as “maybe sometimes?” triggered follow-up prompts offering standardised response options. This mechanism improved scoring accuracy and reduced the risk of misclassification. However, its effectiveness depended on user engagement and could be limited by cognitive load, language barriers, or low responsiveness, highlighting a trade-off between flexibility and robustness in automated screening.

Following completion, the system generated a structured summary comprising item-level scores, overall severity classification, and general resource recommendations. Representative outputs illustrating retrieval-grounded safety handling (verified helpline information in crisis scenarios), clarification of ambiguous input, and PHQ-9 summary generation are shown in Fig 2(b-d). Feedback was designed to be emotionally sensitive and clinically interpretable, with safety guidance grounded in validated psychological resources through the RAG layer. While participants generally found the summaries clear and supportive, the recommendations remained generic and did not incorporate prior psychiatric history or comorbidities, reflecting broader limitations in personalisation within scalable AI-driven screening tools.

### PHQ-9 score concordance

**Fig 3:**
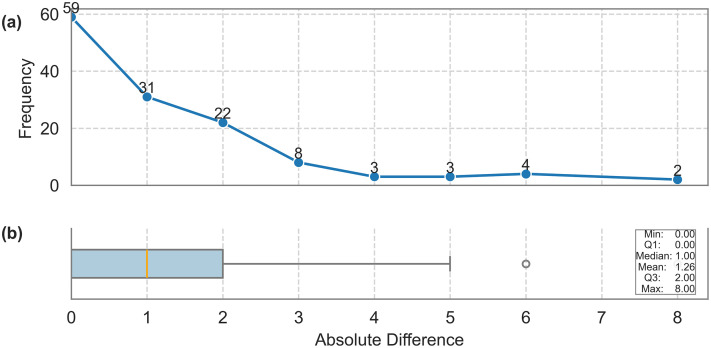
Distribution and variability of absolute differences between self-reported and HopeBot-assisted PHQ-9 scores (n = 132). a, Frequency distribution of absolute differences in PHQ-9 scores between self-administered and HopeBot-assisted assessments. b, Boxplot summarising the range, central tendency, and outliers of absolute differences between the two formats, illustrating that most score discrepancies were small.

A within-subject comparison was conducted to assess agreement between total PHQ-9 scores obtained from the self-administered and HopeBot-assisted formats of the same instrument. As shown in Fig 3, total scores were identical in 59 participants (44.7%). The median absolute difference was 1 point (IQR = 2.00; mean = 1.26), demonstrating high score-level consistency between formats. The distribution of signed differences (median = 0.00; mean = 0.05) indicated no systematic tendency for HopeBot to inflate or reduce severity ratings. A paired Wilcoxon signed-rank test confirmed the absence of directional bias (Z = 1304.0, p = 0.649). Score concordance was further supported by strong association metrics: Spearman’s ρ = 0.92 (p < 0.001) indicated excellent rank-order consistency, while the ICC(3,1) of 0.922 (95% confidence interval [CI] = 0.89–0.94) demonstrated high agreement in absolute score magnitude. Despite this overall concordance, 37 participants (28.0%) shifted into an adjacent PHQ-9 severity category in the HopeBot-assisted version. These shifts occurred primarily between neighbouring categories (e.g., mild to moderate), reflecting minor score variations rather than substantial discrepancies in depressive symptom classification.

**Fig 4:**
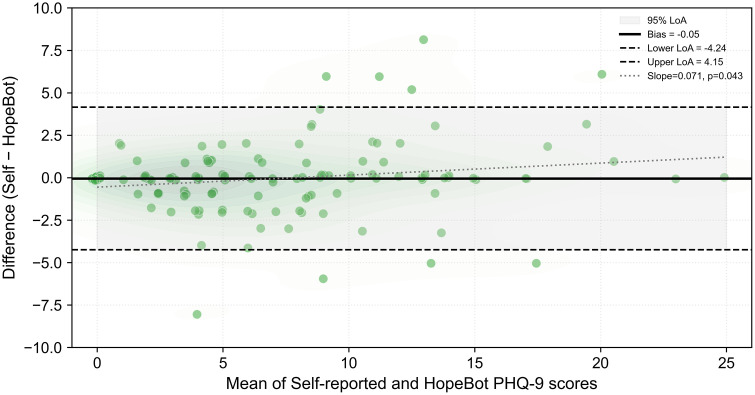
Bland–Altman plot showing agreement between self-reported and HopeBot-assisted PHQ-9 scores (n = 132). The solid line represents the mean difference (bias), and the dashed lines indicate the 95% limits of agreement. Multiple data points overlap due to the discrete 0–27 integer scale of PHQ-9 scoring; slight jitter was applied for visualisation clarity. Differences are centred around zero across the score range.

To further visualise agreement and check for potential proportional bias, a Bland–Altman plot was generated (Fig 4). The mean bias was −0.05, and 95% limits of agreement ranged from −4.24 to 4.15, showing that 95% of paired scores differed by less than ±4 points. A slight positive slope (0.071, p = 0.043) suggested that participants with higher depressive symptom scores tended to report marginally higher self-rated PHQ-9 values, although the effect size was negligible.

To test robustness across participant groups, subgroup analyses were conducted by baseline severity and recruitment country. Concordance remained high in both mild (PHQ-9 < 10: ρ = 0.858, ICC = 0.845) and moderate-to-severe participants (≥10: ρ = 0.711, ICC = 0.785), with no significant difference in absolute score deviation (U = 1579.5, p = 0.219, r = 0.10), indicating a negligible effect size. Across countries, UK participants showed a slightly smaller median deviation (0 vs 1 point; IQR 1.00 vs 2.00) and higher concordance (ρ = 0.938 vs 0.892; ICC = 0.942 vs 0.883). A modest but statistically significant Mann–Whitney difference was observed (U = 2645.0, p = 0.023, r = 0.19). However, the effect size was small, suggesting that the observed difference is unlikely to be clinically meaningful. Overall, these findings support stable performance across cultural and symptom-severity strata.

For the subset who were asked which result they trusted more, 75 participants provided qualitative explanations; of these, 55 (73.3%) had discrepant scores across formats, while 20 (26.7%) gave feedback despite reporting identical scores. The majority (n = 53, 70.7%) expressed greater confidence in the HopeBot-assisted result, 14 (18.7%) preferred their self-assessment, and 8 (10.7%) viewed both as equally valid. Frequencies refer to the number of mentions rather than unique participants, as one participant could contribute to multiple themes. Participants who preferred HopeBot’s result often cited its clearer structure and interpretive scaffolding. The most common rationale (33 mentions, 42.9%) described the chatbot as providing “detailed guidance” or “examples that clarified my emotions”. Others highlighted the emotional support HopeBot offered (15 mentions, 19.5%) or its ability to facilitate deeper self-reflection (8 mentions, 10.4%), contrasting with the quicker, more instinctive nature of the self-test.

Conversely, some participants expressed greater trust in their self-administered PHQ-9 scores. The most frequently coded rationale (8 mentions, 10.4%) described the self-assessment as more intuitive and spontaneous, with several responses noting that the chatbot’s guided prompts occasionally encouraged overthinking. Privacy-related discomfort with disclosing sensitive information to an AI system was also reported (5 mentions, 6.5%). Others pointed to technical limitations (3 mentions, 3.9%), including delays in input recognition or submission issues. One mention (1.3%) described reduced concentration due to the slower pacing of the chatbot interaction.

While a chi-square test showed a significant association between self-reported PHQ-9 severity and trust in HopeBot (χ² = 11.65, df = 4, p = 0.020), this was not supported by logistic regression assuming a linear trend (OR = 1.32, 95% CI 0.79–2.20, p = 0.29), suggesting the relationship may be non-monotonic or driven by specific subgroups.

### Feedback and user experience

Participant feedback, coded by mention frequency, highlighted both strengths and limitations of HopeBot. Personalised advice (50 mentions, 17.9%) was the most frequently cited strength, with participants describing the guidance as specific to their situation and clear in next steps. Emotional support (31 mentions, 11.1%) was characterised as comforting and non-judgmental, while prompt responses (30 mentions, 10.7%) were valued for efficiency and attentiveness. Criticisms focused on generic or shallow replies (33 mentions, 11.8%) that some users found too broad to act on, and on voice-related issues such as delayed output (10 mentions, 3.6%) or mechanical delivery (8 mentions, 2.9%).

During the PHQ-9 screening phase, 79.5% of participants described the transition from open dialogue as natural, and 97.7% found the instructions and questions easy to understand. About one-third (33.3%, n = 44) requested clarification on item interpretation or scoring; among them, 93.2% (n = 41/44) found the chatbot’s explanations helpful. While 77.3% characterised the overall interaction as natural, 17 responses (7.0%) described the transition as abrupt, and 15 (6.1%) mentioned it felt rushed.

**Fig 5:**
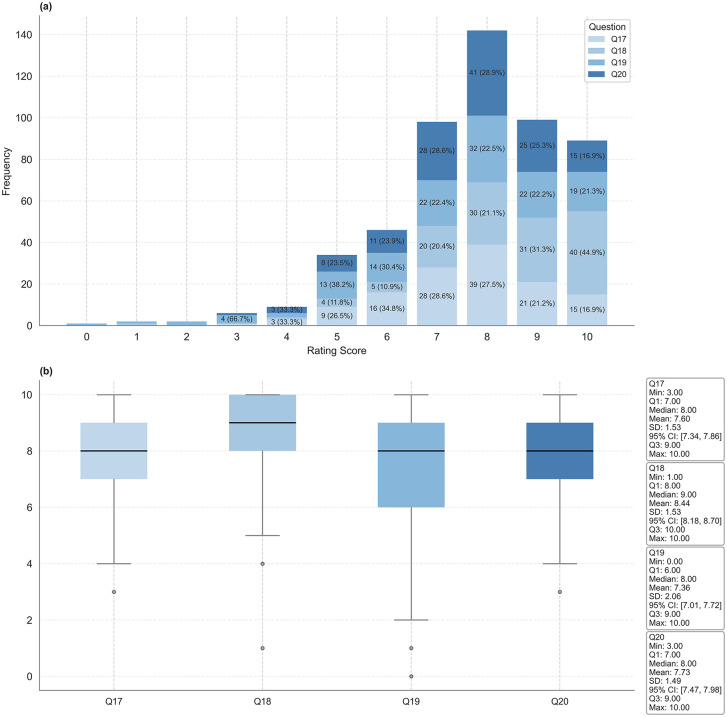
Distribution histograms and boxplots for four HopeBot evaluation items (Q17–Q20). a, Stacked bar plot illustrating the frequency distribution of user ratings (0–10) across four evaluation items. Each bar represents the total number of responses at each score point, subdivided by item. Higher scores indicate more positive user evaluations. Numeric labels within each segment indicate absolute counts and corresponding percentages. To maintain legibility and avoid overcrowding, only segments with sufficient height (e.g., ≥ 2 units) display numeric labels. Low-frequency responses (e.g., ratings 0–4) are fully retained in the underlying analysis but may not be annotated if their bar height falls below the display threshold. b, Boxplots summarising the score distributions for each evaluation item, with accompanying descriptive statistics including minimum, maximum, IQR, median, mean, SD, and 95% CI. These values are displayed to the right of each box for easy comparison. Notes. Q17 = handling of sensitive depression topics; Q18 = comfort expressing feelings without judgment; Q19 = helpfulness of recommendations; Q20 = clarity and tone of voice output.

Quantitative ratings reinforced these observations (Fig 5). On a 10-point scale, participants rated HopeBot’s handling of sensitive topics mean = 7.60, SD = 1.53, with qualitative comments most frequently citing its empathic tone (63 mentions, 31.0%) and practical guidance (50 mentions, 24.6%). However, several participants also described shallow or generic replies (36 mentions, 17.7%), robotic delivery (16 mentions, 7.9%), and repetitive scripted messages (7 mentions, 3.4%). For example, in response to intense emotional disclosures, HopeBot often reiterated that it was not a licensed psychologist and advised users to seek professional help.

HopeBot’s capacity to facilitate emotional expression without judgment received a higher rating (mean = 8.44, SD = 1.53). This was commonly attributed to perceived confidentiality and a non-intrusive communication style. Anonymity was referenced in 72 mentions (38.7%), and 24 mentions (12.9%) highlighted the system’s neutral, non-moralising language.

Perceived usefulness of HopeBot’s recommendations was moderately high (mean = 7.36, SD = 2.06). Many participants described the advice as clear and actionable (72 mentions, 35.0%), whereas 43 mentions (20.9%) characterised it as overly generic or lacking depth. One participant noted that while the guidance was accurate, its similarity to publicly available information reduced its perceived value.

HopeBot’s voice output was rated favourably overall (mean = 7.73, SD = 1.49). Participants frequently highlighted its clear pronunciation (117 mentions, 33.0%) and empathetic, human-like tone (45 mentions, 13.0%), while some noted slow or inaccurate speech recognition (32 mentions, 9.3%) and limited personalisation (25 mentions, 7.2%) as areas for improvement. Despite the positive evaluation of audio quality, nearly half of the participants 45.5% (n = 60), preferred reading the on-screen transcript instead of listening to the full audio, citing greater convenience and discretion. Consistent with this pattern, 51.5% of participants preferred text-based communication, describing it as more practical and private, whereas 40.9% favoured voice interaction for its naturalness and interactivity, and 7.6% expressed no clear preference.

Across all demographic comparisons (Table 2), most associations between participant characteristics and HopeBot ratings were small in magnitude (η² ≤ 0.062; |d| ≤ 0.30) and statistically nonsignificant, indicating limited demographic influence on perceived usability or emotional tone. No significant effects were observed for age, gender, ethnicity, education level, recruitment country (UK vs China), or PHQ-9 severity (both self-reported and HopeBot-assisted) across Q17–Q20. Employment status demonstrated a significant omnibus effect for perceived helpfulness of HopeBot’s recommendations (Q19: F = 3.20, p = .006, η² = 0.133), corresponding to a moderate effect size. However, this effect did not extend to other evaluation domains (Q17, Q18, Q20: p > .25; η² ≤ 0.060), suggesting that employment-related differences were specific to perceived practical utility rather than broader interaction quality.

**Table 2:** Association between demographic characteristics and HopeBot user ratings (Q17–Q20).

Demographic variable	Test	Q17			Q18			Q19			Q20		
		F	p	η²/d	F	p	η²/d	F	p	η²/d	F	p	η²/d
Country of Recruitment (2 levels)	t test	0.535	0.593	0.093	-0.947	0.345	-0.165	-0.598	0.551	-0.104	0.095	0.924	0.017
Age group (6 levels)	ANOVA	0.559	0.731	0.022	1.37	0.241	0.051	1.43	0.219	0.054	1.67	0.147	0.062
Gender (2 levels)	t test	0.696	0.488	0.122	0.186	0.853	0.033	1.72	0.087	0.301	1.22	0.227	0.227
Ethnicity (5 levels)	ANOVA	0.889	0.473	0.027	0.398	0.810	0.012	0.821	0.514	0.025	0.709	0.587	0.022
Highest education (4 levels)	ANOVA	0.174	0.951	0.005	0.831	0.508	0.026	1.18	0.323	0.036	0.607	0.658	0.019
Employment status (6 levels)	ANOVA	0.661	0.681	0.031	1.32	0.253	0.060	3.20	**0.006**	0.133	1.18	0.320	0.054
Mental health support experience (binary)	t test	-0.637	0.528	-0.143	-1.53	0.137	-0.457	-2.65	**0.012**	-0.632	-1.14	0.261	-0.222
Chatbot experience (binary)	t test	-1.44	0.153	-0.267	0.380	0.705	0.071	-0.859	0.392	-0.159	0.134	0.894	0.025
PHQ-9 Severity Result – HopeBot (5 levels)	ANOVA	1.23	0.300	0.037	0.462	0.763	0.014	1.57	0.188	0.047	1.72	0.150	0.051
PHQ-9 Severity Result – Self-test (5 levels)	ANOVA	1.40	0.238	0.042	0.284	0.888	0.009	0.823	0.513	0.025	0.394	0.813	0.012

Univariable analyses were conducted to examine associations between participant demographic characteristics and user evaluation ratings. Independent-samples t tests were applied to binary variables, and one-way ANOVA was used for variables with more than two levels. Effect sizes are reported as Cohen’s d for t tests and η² for ANOVA to quantify the magnitude of associations. Statistically significant p values (p < 0.05) are shown in bold.

Notes. Q17 = handling of sensitive depression topics; Q18 = comfort expressing feelings without judgment; Q19 = helpfulness of recommendations; Q20 = clarity and tone of voice output. All values are rounded to three significant figures. Bold indicates p < 0.05.

Participants with prior experience of mental health treatment reported lower helpfulness ratings (Q19: t = –2.65, p = .012, d = –0.63), reflecting a moderate difference in perceived recommendation value. In contrast, ratings for handling sensitive topics (Q17), emotional comfort (Q18), and voice clarity (Q20) did not differ by treatment history (p ≥ .10; |d| ≤ 0.46). Previous use of mental health chatbots was not associated with any rating outcome (all p ≥ .15; |d| ≤ 0.27). Overall, feedback patterns were broadly consistent across demographic and cultural subgroups.

### Perceived acceptability and adoption intentions

Participant preferences for PHQ-9 administration formats varied. A majority (n = 92, 69.7%) indicated a preference for the chatbot-assisted format over self-completion, citing that it felt more engaging (39 mentions, 20.2%), emotionally supportive and interactive (36 mentions, 18.7%), and provided real-time clarification and guidance during question interpretation (32 mentions, 16.6%). In contrast, participants who preferred self-administration emphasised its efficiency (20 mentions, 10.4%) and its suitability for situations without immediate emotional distress (13 mentions, 6.7%). A small subset (n = 6, 4.6%) expressed no clear preference. Preference was associated with employment status overall (χ² = 21.69, df = 12, p = .041), but no individual subgroup (all p > .5) showed a statistically significant odds ratio, suggesting weak or diffuse effects.

Among all respondents, only 19.7% (n = 26) reported prior engagement with professional mental-health services, yet all participants were invited to reflect on HopeBot’s perceived performance relative to human counselling. Consistent with earlier themes, participants positively appraised the chatbot’s structured questioning and empathic tone (each 38 mentions, 12.3%). However, limitations were frequently noted, including perceptions of insufficient human-likeness (58 mentions, 18.7%), emotional shallowness or detachment (42 mentions, 13.5%), and overly generic or impersonal responses lacking individual tailoring (19 mentions, 6.1%).

Regarding adoption, most participants (n = 115, 87.1%) indicated willingness to use or recommend HopeBot in the future. Many perceived its potential to support mental health screening, particularly through early detection and emotionally responsive communication (56 mentions, 22.0%; 32 mentions, 12.6%). Other cited advantages included immediate accessibility (15 mentions, 5.9%), rapid response time (14 mentions, 5.5%), and anonymous interaction (8 mentions, 3.1%). Several respondents (n = 12, 4.7%) emphasised that such tools should complement rather than replace professional care. Concerns focused on the need for clinical validation (4 mentions, 1.6%), possible diagnostic unreliability (3 mentions, 1.2%), and data privacy risks (11 mentions, 4.4%). Nonetheless, willingness to recommend was significantly lower among participants with prior mental health service experience (Fisher’s exact OR = 0.22, CI 0.05–0.92, p = .0497).

## Discussion

### Principal findings

This study demonstrates the feasibility of administering the PHQ-9 through a GPT-4o-powered, voice-interactive chatbot (HopeBot). HopeBot-assisted and self-administered scores showed high concordance (ICC = 0.92; median absolute difference = 1 point), supporting its feasibility as a reliable administration method. Participants generally found the chatbot easy to use and responsive, consistent with usability ratings and suggesting that conversational delivery can improve engagement within standardised screening frameworks. Importantly, the PHQ-9 served as a validated screening framework for evaluating format-level consistency in administration rather than as a diagnostic gold standard, which remains clinician-administered interviews.

Although clinician-administered PHQ-9 interviews detect suicidality and comorbid conditions with higher sensitivity [15], self-administered formats remain standard in digital screening, showing acceptable psychometric performance (sensitivity ≈0.80; specificity ≈0.85) [11,61]. The self-report version was therefore used as a pragmatic comparator, reflecting real-world contexts in which individuals typically complete questionnaires independently. Within this framework, the observed concordance between HopeBot-assisted and self-administered PHQ-9 scores supports the format-level fidelity of LLM-assisted administration. However, further validation against clinician-led diagnostic interviews is required to establish diagnostic accuracy and clinical applicability.

Beyond score concordance, HopeBot elicited considerable user trust. Among the 75 participants who directly compared the two formats, 70.7% (n = 53/75) expressed greater confidence in the chatbot-assisted scores, attributing this preference to features such as real-time clarification (33 mentions, 42.9%) and an empathic tone (15 mentions, 19.5%). These interactional features partially resembled elements of semi-structured interviews, enhancing engagement and clarity while preserving the scalability and standardisation of automated delivery.

Perceptions of recommendation helpfulness (Q19) varied across subgroups. Full-time students and individuals managing household responsibilities gave lower ratings than full-time employees (F = 3.20, p = .006, η² = 0.133), and those with prior mental health service experience rated recommendations less helpful than first-time users (t = –2.65, p = .012, d = –0.63). These differences likely reflect heightened expectations shaped by users’ life context and therapeutic background. Students and homemakers—often managing complex emotional demands with limited external support—may have anticipated greater empathy and personalisation [62,63]. Similarly, individuals with prior counselling experience may have evaluated responses against professional standards [7], consistent with expectancy-disconfirmation theory [64]. These findings suggest that trust and perceived usefulness of AI-based mental-health tools are shaped by users’ experiential backgrounds, underscoring the need for adaptive designs that accommodate diverse expectations of empathy and personalisation.

### Comparison with prior systems

As summarised in Table 3, HopeBot introduces several substantive advancements over earlier PHQ-9 chatbots that rely primarily on Dialogflow-based intent matching (e.g., Perla [18], Marcus [27], DEPRA [21]). By integrating RAG with the GPT-4o architecture, HopeBot supports fully open-ended interaction while meeting the technical constraints of real-time screening. GPT-4o was selected based on three key considerations: (1) independent benchmarks reported the lowest latency among publicly available LLMs (≈0.45 s for text, ≈ 0.32 s for audio) at that time, outperforming Claude 3 and Gemini 1.5 [66];(2) its unified multimodal framework eliminates the need for separate ASR–TTS pipelines, which remain necessary for open-source and contemporary commercial alternatives [67]; and (3) its extended context window (100k tokens) and multilingual tokeniser ensure compatibility with the demands of interactive PHQ-9 delivery while preserving clinical safety constraints [68]. Although LLMs such as Claude 3, Gemini 1.5, and Llama-3 warrant future evaluation, their higher latency and limited support for speech and alignment control made them unsuitable for the present implementation.

**Table 3:** Comparison of automated depression screening tools across key functional dimensions.

Dimension	Core architecture	Language flexibility	Screening instrument	Explainability	User study (N)	Empathy/tone	Deployment potential	Key limitation in prior work
Perla (2020) [18]	Google Dialogflow with ML-based intent classification and Firebase backend	Natural language input with ~200 phrases per item and synonym matching	PHQ-9 (Spanish)	Provides total score, risk status, and resource links at the end	276 participants; 108 completed both Perla and web-based PHQ-9	Supportive tone with female persona and encouraging prompts	Web and major messaging platforms (e.g., Messenger, Google Assistant, Telegram)	Limited validation, English-only tools, and low engagement in prior form-based tools
Marcus (2023) [27]	Dialogflow intents + BERT model (Node.js/ GCP; Kommunicate UI)	Free-text input classified to PHQ-9	PHQ-9 (English)	Outputs total PHQ-9 score only	81 U.S. college students (130 enrolled)	Neutral; static male avatar; no empathy modelling	iOS app and web chat prototype	Earlier, PHQ-9 chatbots lacked validation with U.S. college students and used only fixed-choice input
IGOR (2021) [26]	Dialogflow intents with Node.js + Firebase backend	Button/option input (PHQ-9 scores 0–3)	PHQ-9	Sends total score to clinician; not shown to user	10 university staff (usability test)	Neutral; no empathic responses	Prototype within MS self-management app	Results hidden from the user; rule-based flow fails on off-topic input
DEPRA (2023) [21]	Dialogflow chatbot with 27 SIGH-D/IDS-C intents	Open-text input with intent matching	SIGH-D + IDS-C	Final score and severity level only; no item-level feedback	50 Australian adults	Neutral tone; no empathy modelling	Facebook Messenger chatbot prototype	High cognitive load; long completion time
EmoScan (2024) [28]	Mistral-7B fine-tuned on synthetic clinical interviews (PsyInterview)	Free-text, multi-turn inputs; fine-tuned LLM	DSM-5-based emotional disorder classification (coarse & fine-grained)	LLM-generated explanations based on DSM-5 criteria	1,157 synthetic cases; 50 expert-evaluated; GPT-4-based performance evaluation	Simulated empathy assessed by GPT-4 and clinical experts	Research prototype; not deployed clinically	Heavily synthetic Reliance on synthetic data; limited real-world generalisability
Moodpath App (2021) [65]	Smartphone app, 3 × daily AA (45 ICD-10 items + mood)	Tap yes/no → 4-level severity; 5-point mood scale	DSM-5-based emotional disorder classification (coarse & fine-grained)	14-day summary with score, severity band & mood charts	113 general-population users	Neutral; no empathic dialogue	Live on iOS & Android	Prior tools used retrospective questionnaires with little validation
HopeBot (2025)	GPT-4o with RAG	Supports open-ended user input in various languages	PHQ-9 administered via free-text/voice dialogue, with dynamic clarification and fuzzy score interpretation	Provides item-level rationales and context-relevant evidence drawn from curated knowledge bases	132 participants (Chinese + UK residents)	LLM-generated responses were generally perceived as empathic; mean comfort rating 8.51/10, though variation in emotional tone and delivery style was reported	Available via web interface; compatible with both desktop and mobile devices (iOS, Android); supports both Mandarin and English voice/text modalities	Absence of non-verbal cues, occasional mechanical tone in voice output, and lack of clinical validation for diagnostic reliability

This architecture enables dynamic turn-taking, clarification of ambiguous responses, and seamless support for languages beyond English and Mandarin—capabilities not reported in prior systems. Transparency is further enhanced through item-level scoring and source-linked rationales, features absent in comparators such as IGOR [26] and Marcus [27]. Among participants who engaged with the clarification module, 93.2% indicated that these explanations improved their response accuracy.

While HopeBot was not designed to substitute for clinician-led diagnosis, its findings indicate potential utility as a preliminary engagement or triage aid within mental-health screening workflows [69]. Unlike earlier rule-based systems that restricted user expression [70], HopeBot’s adaptive clarification maintains alignment with the PHQ-9 item structure while allowing users to articulate symptoms more naturally, which may enhance clarity and perceived relevance.

In practical terms, this standardised yet conversational approach may support early identification of depressive features, particularly among individuals who might otherwise defer or avoid assessment. The high concordance with self-administered PHQ-9 scores (ICC = 0.92) suggests that conversational delivery can preserve quantitative integrity while improving accessibility and engagement. Collectively, these characteristics suggest that HopeBot could function as a research-oriented adjunct bridging self-report and clinician-administered formats, providing a framework for scalable and low-risk screening.

User feedback provides further support for these procedural benefits. In contrast to Marcus, where only 18.1% of users preferred the chatbot over conventional self-report [27], 69.7% (n = 92) of participants in this study favoured HopeBot, and 87.1% (n = 115) expressed willingness to reuse or recommend it. These results indicate higher levels of user acceptance and engagement than those reported for earlier rule-based systems. Combined with its low-latency performance, bilingual adaptability, and transparent item-level explanations, this evidence provides preliminary, exploratory support for HopeBot’s feasibility and user alignment within future digital screening research workflows.

From a practical deployment perspective, the PHQ-9 remains highly efficient in self-administered form, typically requiring approximately 2–3 minutes to complete [71]. By contrast, internal pilot testing of HopeBot indicated a substantially longer interaction time. Across ten internal test sessions, a complete HopeBot interaction, including open-ended dialogue and PHQ-9 administration, lasted a mean of 25 minutes and 23 seconds, with duration varying according to the extent of user self-disclosure and conversational depth.

This increased time investment reflects the system’s design emphasis on open-ended dialogue, clarification, and emotional support rather than rapid item completion. While less suitable for high-throughput screening, such an approach may be justified in contexts where engagement, comprehension, or psychological reassurance are prioritised, including remote or underserved settings, populations with low mental health literacy, or individuals reluctant to complete standard questionnaires. In these scenarios, conversational screening may complement brief self-report instruments rather than replace them.

In terms of deployment requirements, HopeBot does not require specialised hardware on the user side, as computation is handled via cloud-based language model inference and lightweight vector retrieval. Operational costs are therefore primarily associated with API usage (including language model inference and optional speech transcription and synthesis), alongside standard hosting and data management. These costs can be moderated through design choices such as default text-based interaction, constrained retrieval context, and session-length limits, depending on deployment setting and scale.

### Technical, ethical, and linguistic constraints

Despite its technical strengths, HopeBot did not fully replicate the relational depth of professional counselling. A substantial number of participants described the interaction as emotionally flat or impersonal (58 mentions, 18.7%), citing insufficient affective nuance (42 mentions, 13.5%) and reliance on generic responses (19 mentions, 6.1%). These limitations indicate that, even with prompt tuning and retrieval augmentation, simulated empathy remains perceptibly artificial. These limitations underscore the persistent gap between linguistic fluency and emotional authenticity in LLM-mediated dialogue.

More broadly, these limitations reflect structural constraints inherent in current-generation conversational AI [72]. While current LLMs can generate contextually coherent text, they lack access to non-verbal cues such as tone, expression, or posture that clinicians rely on to assess distress [22]. Cross-linguistic speech synthesis poses further challenges. Although GPT-4o natively supports Mandarin text, the deployment of a text-to-speech engine optimised for English prosody introduced prosodic inconsistencies that reduced perceived naturalness and constrained affective expressiveness [73]. Future developments in culturally adaptive voice models may improve emotional fidelity across languages.

Beyond technical issues, ethical and epistemic considerations remain. Repeated exposure to polished AI language may subtly influence how users articulate emotional experiences [74]. Clinicians may also adjust judgments when confronted with opaque AI recommendations, even in the absence of a clear clinical rationale [75], raising concerns about automation bias and the erosion of clinical autonomy. Accordingly, systems such as HopeBot should be positioned as adjunctive tools that support, rather than replace, professional expertise [22]. Responsible deployment will require transparent algorithmic logic, explicit clinical oversight, and safeguards that ensure interpretability, accountability, and informed consent. Future research should extend beyond screening accuracy to investigate how such technologies influence therapeutic relationships, user trust, and long-term mental health outcomes.

### Study limitations

Several methodological and contextual limitations warrant acknowledgement. First, no clinician-administered diagnostic benchmark was used in the present study. The validation framework therefore compared HopeBot-assisted and self-administered PHQ-9 formats rather than clinician-led references; results reflect format-level concordance in administration rather than diagnostic validity. Second, the sequential completion of both PHQ-9 versions within one session may have introduced recall bias; counterbalanced or time-separated designs should be explored. Third, the sample skewed younger and digitally literate, which may overestimate usability in older or digitally excluded groups. Fourth, although participants were recruited from both the UK and China, the study was not designed or powered to formally evaluate cultural, linguistic, or cross-national differences in interaction patterns or user perceptions. Accordingly, subgroup analyses were exploratory and should not be interpreted as evidence of cross-cultural equivalence. Fifth, while both quantitative and qualitative subgroup comparisons were conducted, some demographic strata (e.g., ethnic minority or severe-symptom groups) were small, limiting the statistical power for detecting fine-grained effects. In addition, the controlled testing environment may not reflect naturalistic conditions. All findings are specific to GPT-4o and may not extend to other LLM-based systems.

### Future research

Future research should evaluate clinical utility, safety, and equity across health-care settings. Multisite and randomised trials comparing LLM-assisted, self-administered, and clinician-administered PHQ-9 formats would clarify diagnostic performance and workflow impact. Development of governance frameworks, including escalation protocols, audit trails, and transparent disclosures, will be essential for regulatory compliance. Particular attention is needed for high-risk encounters requiring non-verbal cue interpretation and for underserved populations with limited digital access. Cross-cultural extensions, already preliminarily supported in this cohort, should be validated across health systems in both the UK and China.

## Conclusion

HopeBot feasibly administers the PHQ-9 with high scoring fidelity and user acceptability. By integrating conversational empathy with validated screening logic, it may serve as a scalable adjunct for early depression detection. Continued evaluation across clinical and cultural contexts will be essential to ensure responsible and equitable translation into practice.

## Supporting information

S1 Table GRRAS-checklist. (DOCX)

S2 Table STARD2015 Checklist. (DOCX)

S3 Table PHQ-9 score differences and subgroup analysis between HopeBot-assisted and self-administered assessments across demographic and experience variables. (DOCX)

S4 Table Distribution and summary of thematic codes for participant responses to open-ended survey questions (Q8–Q18). (DOCX)

S5 Table Distribution and summary of thematic codes for participant responses to open-ended survey questions (Q19–Q25). (DOCX)

S1 Text Core system prompt used in the study. (DOCX)

S2 Text Ethics, safety, and privacy. (DOCX)

S3 Text 25 item questionnaire. (DOCX)

S4 Text Codebook. (DOCX)
