# Supplementary material for: Feasibility and user evaluation of HopeBot: An LLM-powered conversational chatbot for depression screening
Source: PLOS Digit Health. 2026 Jun 25;5(6):e0001446. doi: 10.1371/journal.pdig.0001446 (PMC13298971; doi:10.1371/journal.pdig.0001446)
Supplement: S1 Table — (DOCX) [file pdig.0001446.s001.docx]

**Supporting information**

**S1 Table.** [**GRRAS-checklist**](https://sites.usp.br/praticaempesquisa/wp-content/uploads/sites/1049/2021/11/GRRAS-checklist.doc).

| **Section** | **Item #** | **Checklist item** | **Reported on page #** |
| --- | --- | --- | --- |
| Title/Abstract | 1 | Identify in title or abstract that interrater/intrarater  reliability or agreement was investigated. | 1-2 |
| Introduction | 2 | Name and describe the diagnostic or measurement device of interest explicitly. | 3-4 |
|  | 3 | Specify the subject population of interest. | 3-4 |
|  | 4 | Specify the rater population of interest (if applicable). | 3-4 |
|  | 5 | Describe what is already known about reliability and  agreement and provide a rationale for the study (if applicable). | 4-5 |
| Methods | 6 | Explain how the sample size was chosen. State the determined number of raters, subjects/objects, and replicate observations. | 9 |
|  | 7 | Describe the sampling method. | 9 |
|  | 8 | Describe the measurement/rating process (e.g. time interval between repeated measurements, availability  of clinical information, blinding). | 8-9 |
|  | 9 | State whether measurements/ratings were conducted independently. | 9 |
|  | 10 | Describe the statistical analysis. | 9-10 |
| Results | 11 | State the actual number of raters and subjects/objects  which were included and the number of replicate observations which were conducted. | 11&12 |
|  | 12 | Describe the sample characteristics of raters and  subjects (e.g. training, experience). | 11-12 |
|  | 13 | Report estimates of reliability and agreement including measures of statistical uncertainty. | 13-14 |
| Discussion | 14 | Discuss the practical relevance of results. | 18-20 |
| Auxiliary material | 15 | Provide detailed results if possible (e.g. online). | 23-24 |
