# Supplementary material for: Feasibility and user evaluation of HopeBot: An LLM-powered conversational chatbot for depression screening
Source: PLOS Digit Health. 2026 Jun 25;5(6):e0001446. doi: 10.1371/journal.pdig.0001446 (PMC13298971; doi:10.1371/journal.pdig.0001446)
Supplement: S2 Table — (DOCX) [file pdig.0001446.s002.docx]

**Supporting information**

**S2 Table. STARD2015 Checklist.**

|  | **Section & Topic** | **No** | **Item** | **Reported on page #** |
| --- | --- | --- | --- | --- |
|  |  |  |  |  |
|  | **TITLE OR ABSTRACT** |  |  |  |
|  |  | 1 | Identification as a study of diagnostic accuracy using at least one measure of accuracy  (such as sensitivity, specificity, predictive values, or AUC) |  |
|  | **ABSTRACT** |  |  |  |
|  |  | 2 | Structured summary of study design, methods, results, and conclusions  (for specific guidance, see STARD for Abstracts) | 1-2 |
|  | **INTRODUCTION** |  |  |  |
|  |  | 3 | Scientific and clinical background, including the intended use and clinical role of the index test | 3-4 |
|  |  | 4 | Study objectives and hypotheses | 4-5 |
|  | **METHODS** |  |  |  |
|  | **Study design** | 5 | Whether data collection was planned before the index test and reference standard  were performed (prospective study) or after (retrospective study) | 9 |
|  | **Participants** | 6 | Eligibility criteria | 9 |
|  |  | 7 | On what basis potentially eligible participants were identified  (such as symptoms, results from previous tests, inclusion in registry) |  |
|  |  | 8 | Where and when potentially eligible participants were identified (setting, location and dates) | 9 |
|  |  | 9 | Whether participants formed a consecutive, random or convenience series | 9 |
|  | **Test methods** | 10a | Index test, in sufficient detail to allow replication | 5 |
|  |  | 10b | Reference standard, in sufficient detail to allow replication | 6 |
|  |  | 11 | Rationale for choosing the reference standard (if alternatives exist) | 5-6 |
|  |  | 12a | Definition of and rationale for test positivity cut-offs or result categories  of the index test, distinguishing pre-specified from exploratory |  |
|  |  | 12b | Definition of and rationale for test positivity cut-offs or result categories  of the reference standard, distinguishing pre-specified from exploratory |  |
|  |  | 13a | Whether clinical information and reference standard results were available  to the performers/readers of the index test |  |
|  |  | 13b | Whether clinical information and index test results were available  to the assessors of the reference standard |  |
|  | **Analysis** | 14 | Methods for estimating or comparing measures of diagnostic accuracy | 10 |
|  |  | 15 | How indeterminate index test or reference standard results were handled |  |
|  |  | 16 | How missing data on the index test and reference standard were handled | 10 |
|  |  | 17 | Any analyses of variability in diagnostic accuracy, distinguishing pre-specified from exploratory | 9-10 |
|  |  | 18 | Intended sample size and how it was determined | 9 |
|  | **RESULTS** |  |  |  |
|  | **Participants** | 19 | Flow of participants, using a diagram |  |
|  |  | 20 | Baseline demographic and clinical characteristics of participants | 11-12 |
|  |  | 21a | Distribution of severity of disease in those with the target condition | 12 |
|  |  | 21b | Distribution of alternative diagnoses in those without the target condition | 12 |
|  |  | 22 | Time interval and any clinical interventions between index test and reference standard |  |
|  | **Test results** | 23 | Cross tabulation of the index test results (or their distribution)  by the results of the reference standard | 13-14 |
|  |  | 24 | Estimates of diagnostic accuracy and their precision (such as 95% confidence intervals) | 13-14 |
|  |  | 25 | Any adverse events from performing the index test or the reference standard |  |
|  | **DISCUSSION** |  |  |  |
|  |  | 26 | Study limitations, including sources of potential bias, statistical uncertainty, and generalisability | 22 |
|  |  | 27 | Implications for practice, including the intended use and clinical role of the index test | 20-22 |
|  | **OTHER INFORMATION** |  |  |  |
|  |  | 28 | Registration number and name of registry | 2&5 |
|  |  | 29 | Where the full study protocol can be accessed | 5 |
|  |  | 30 | Sources of funding and other support; role of funders | 24 |
|  |  |  |  |  |
