# Supplementary material for: Feasibility and user evaluation of HopeBot: An LLM-powered conversational chatbot for depression screening
Source: PLOS Digit Health. 2026 Jun 25;5(6):e0001446. doi: 10.1371/journal.pdig.0001446 (PMC13298971; doi:10.1371/journal.pdig.0001446)
Supplement: S3 Table — (DOCX) [file pdig.0001446.s003.docx]

**Supporting information**

**S3 Table.PHQ-9 score differences (HopeBot – Self-administered) across demographic and experience variables.**

| **Variable** | **Group** | **Median** | **IQR** | **Test** | **Statistic** | **p_raw** | **p_holm** |
| --- | --- | --- | --- | --- | --- | --- | --- |
| **Age group (years)** | 18–24 years old | 0.00 | 2.00 | Kruskal–Wallis H | 6.565 | 0.766 | 1.000 |
|  | 25–34 years old | 0.00 | 0.25 |  |  |  |  |
|  | 35–44 years old | 0.00 | 0.75 |  |  |  |  |
|  | 45–54 years old | 0.00 | 3.50 |  |  |  |  |
|  | 55–64 years old | 0.00 | 1.00 |  |  |  |  |
|  | 65–70 years old | 0.00 | 2.00 |  |  |  |  |
| **Gender** | Female | 0.00 | 1.00 | Mann–Whitney U | 2243.5 | 0.690 | 1.000 |
|  | Male | 0.00 | 2.00 |  |  |  |  |
| **Ethnicity** | Asian or Asian British | 0.00 | 2.00 | Kruskal–Wallis H | 4.646 | 0.326 | 1.000 |
|  | Black / Black British / Caribbean | 0.50 | 2.00 |  |  |  |  |
|  | Mixed or Multiple groups | 0.50 | 0.50 |  |  |  |  |
|  | White | 0.00 | 0.00 |  |  |  |  |
|  | Prefer not to say | 3.00 | 0.00 |  |  |  |  |
| **Highest education** | No formal | 0.00 | 0.00 | Kruskal–Wallis H | 2.140 | 0.952 | 1.000 |
|  | Further education | 0.50 | 1.25 |  |  |  |  |
|  | Undergraduate degree | 0.00 | 1.25 |  |  |  |  |
|  | Postgraduate degree | 0.00 | 0.00 |  |  |  |  |
|  | Prefer not to say | 0.00 | 0.00 |  |  |  |  |
| **Employment status** | Full-time employment | 0.00 | 1.00 | Kruskal–Wallis H | 8.130 | 0.616 | 1.000 |
|  | Part-time employment | 0.00 | 0.50 |  |  |  |  |
|  | Full-time education / training | 0.00 | 0.00 |  |  |  |  |
|  | Looking after the home | 0.00 | 0.00 |  |  |  |  |
|  | Retired | 1.00 | 1.00 |  |  |  |  |
|  | Home | −2.00 | 1.50 |  |  |  |  |
|  | Other | 0.00 | 0.50 |  |  |  |  |
|  | Prefer not to say | −1.00 | 0.00 |  |  |  |  |
|  | Student | 0.00 | 3.00 |  |  |  |  |
| **Familiarity with LLMs** | HEARD_ONLY | 1.00 | 1.00 | Kruskal–Wallis H | 13.313 | 0.021 | 0.165 |
|  | NO_EXPERIENCE | −3.00 | 3.00 |  |  |  |  |
|  | OCCASIONAL_USER | −1.00 | 2.25 |  |  |  |  |
|  | REGULAR_USER | 0.00 | 1.00 |  |  |  |  |
|  | TECHNICAL_EXPERT | 0.00 | 0.50–7.00 |  |  |  |  |
| **Mental health chatbot experience** | No | 0.00 | 1.00 | Mann–Whitney U | 1818.0 | 0.646 | 1.000 |
|  | Yes | 0.00 | 2.00 |  |  |  |  |
| **Previous mental health support experience** | No | 0.00 | 2.00 | Mann–Whitney U | 1112.0 | 0.111 | 0.774 |
|  | Yes | 0.00 | 1.75 |  |  |  |  |

Group-wise median and interquartile range (IQR) of PHQ-9 score differences (HopeBot – Self-administered) by demographic and experience variables.

For each subgroup, medians and IQRs are presented together with the corresponding nonparametric test statistics. Mann–Whitney U tests were used for binary variables and Kruskal–Wallis H tests for multi-category variables. P values were corrected for multiple comparisons using the Holm method.

No subgroup differences reached statistical significance after correction (all p > 0.05), suggesting that the differences between HopeBot-assisted and self-administered PHQ-9 scores were stable across age, gender, ethnicity, education, employment, familiarity with large language models (LLMs), and prior mental-health-related experiences.
