# Supplementary material for: Feasibility and user evaluation of HopeBot: An LLM-powered conversational chatbot for depression screening
Source: PLOS Digit Health. 2026 Jun 25;5(6):e0001446. doi: 10.1371/journal.pdig.0001446 (PMC13298971; doi:10.1371/journal.pdig.0001446)
Supplement: S4 Table — (DOCX) [file pdig.0001446.s004.docx]

**S4 Table. Distribution of thematic codes for participant responses to open-ended survey questions (Q8–Q18).**


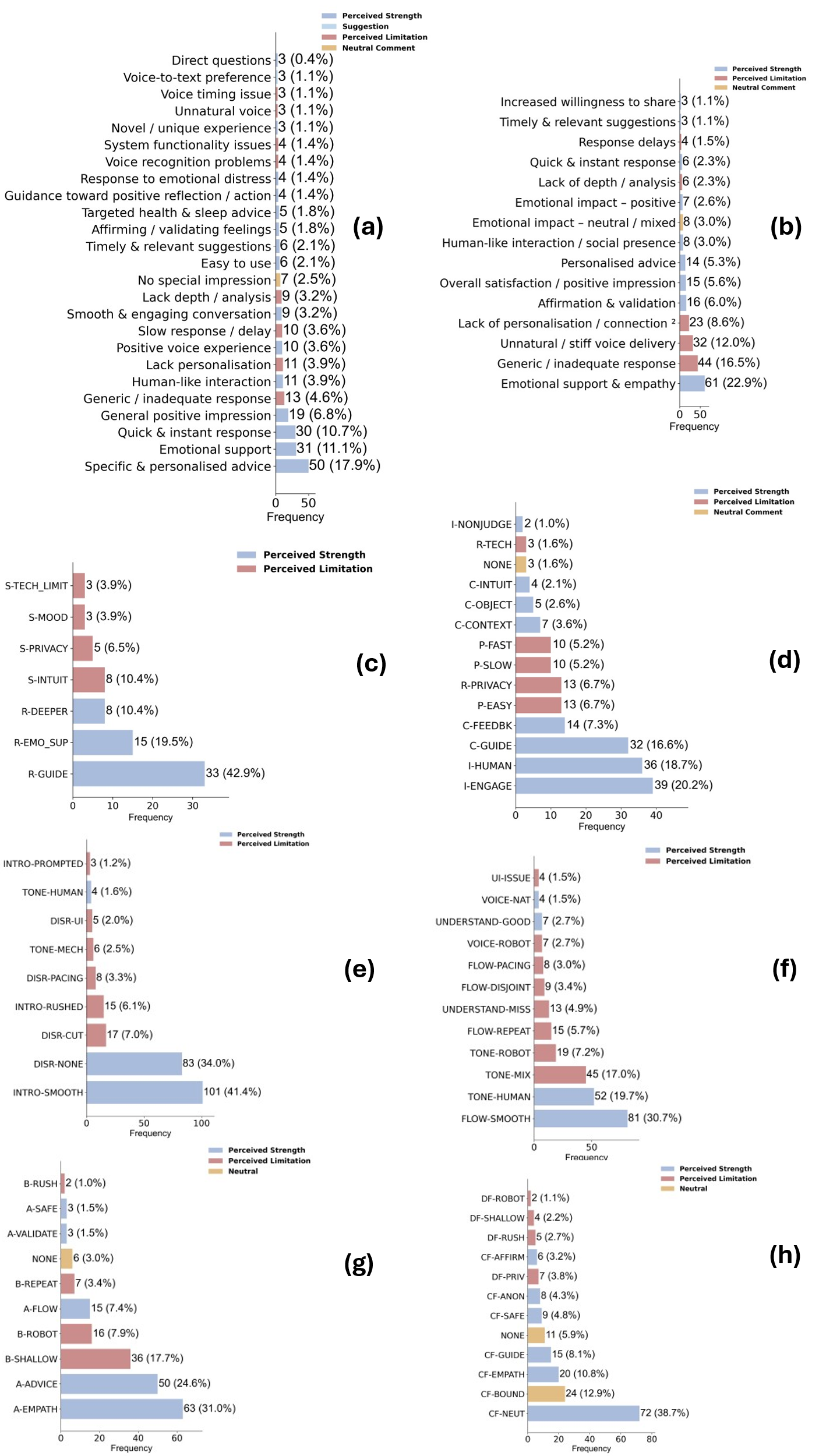


This composite figure visualises eight subplots (a–h), each corresponding to a specific open-ended question from the HopeBot user study. Horizontal bar charts illustrate the frequency and proportion of thematically coded responses. To enhance visual clarity, only codes that occurred at least twice (n ≥ 2) are shown; responses coded fewer than twice were excluded from the plots but were included in percentage calculations to ensure consistency with reported results.

Bars are colour-coded to reflect the nature of user feedback:

• Perceived Strength
• Perceived Limitation
• Suggestion
• Neutral/Conditional Comment

a, Q8 – Was there anything that stood out to you during your interaction with HopeBot?

b, Q9 – Did you feel understood by HopeBot, or did it ever feel robotic?

c, Q12 – Is there a difference between your self-assessed PHQ-9 score and the HopeBot-assisted one? Which results do you find more trustworthy?

d, Q13 – How does using HopeBot to complete the PHQ-9 compare to completing it alone at home? Which did you prefer?

e, Q14 – Did HopeBot introduce the PHQ-9 naturally or disruptively?

f, Q16 – Did the overall conversation with HopeBot feel natural or robotic?

g, Q17 – How well did HopeBot handle sensitive depression-related topics (1–10)?

h, Q18 – How comfortable did you feel expressing your feelings without judgment (1–10)?

Proportions (in parentheses) were calculated based on the total number of valid responses per item (see Methods). This figure supplements the qualitative findings by illustrating the distribution and relative weight of user experiences across key domains of chatbot interaction.
