# Supplementary material for: Feasibility and user evaluation of HopeBot: An LLM-powered conversational chatbot for depression screening
Source: PLOS Digit Health. 2026 Jun 25;5(6):e0001446. doi: 10.1371/journal.pdig.0001446 (PMC13298971; doi:10.1371/journal.pdig.0001446)
Supplement: S5 Table — (DOCX) [file pdig.0001446.s005.docx]

**S5 Table. Distribution of thematic codes for participant responses to open-ended survey questions (Q19–Q25).**

**
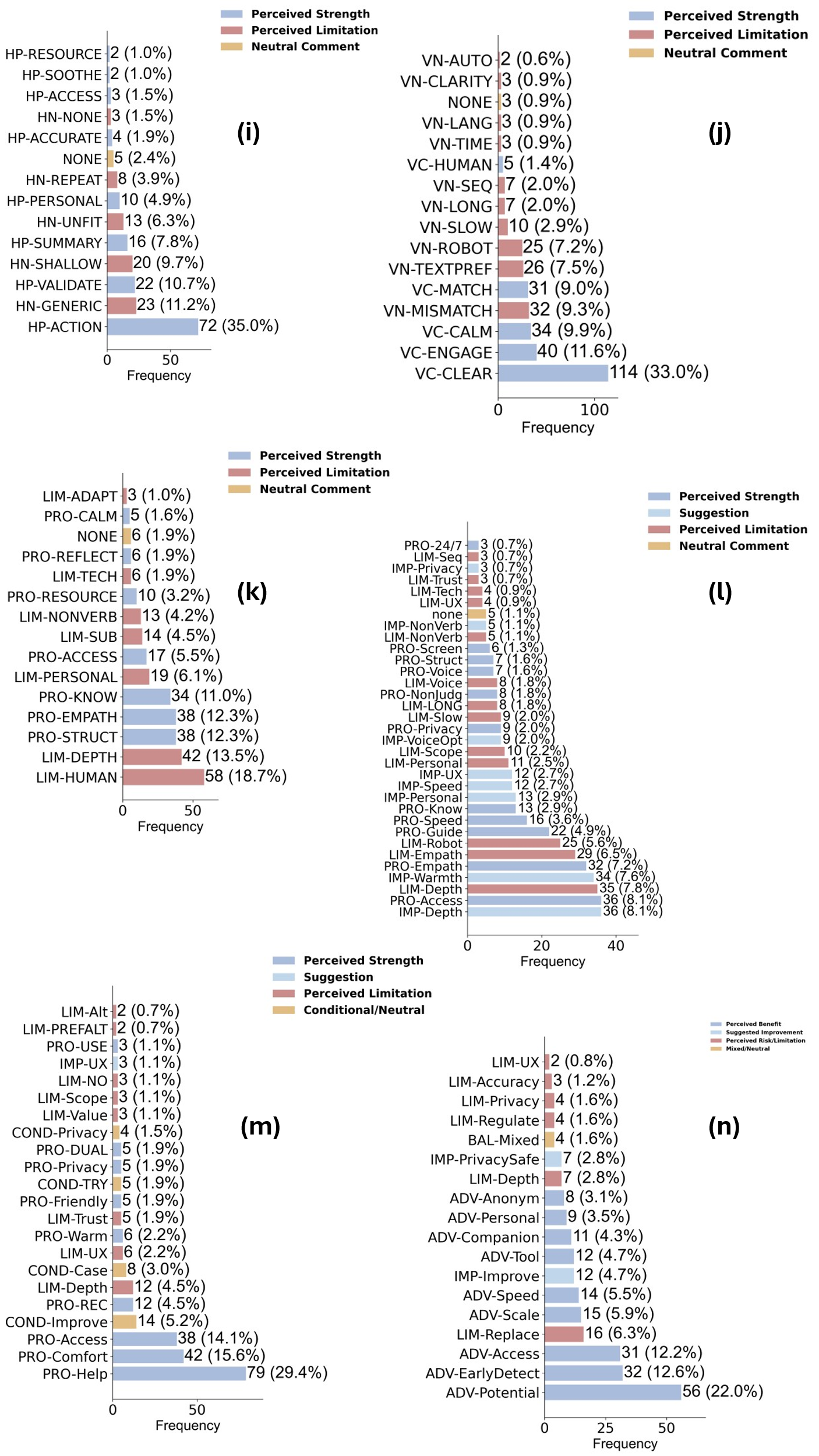
**

This composite figure presents six horizontal bar charts (i–n), each corresponding to a distinct open-ended question from the HopeBot user study (Q19–Q25). For each item, thematically coded responses are visualised in terms of frequency and percentage, with colour-coding to indicate the nature of user feedback:

• Perceived Strength
• Perceived Limitation
• Suggestion
• Neutral/Conditional Comment

To optimise clarity, only codes that occurred at least twice (n ≥ 2) are displayed in the plots; codes with fewer than two occurrences are omitted from the bars but included in percentage calculations to ensure consistent denominators with the main results. Proportions in parentheses reflect the share of total valid responses for each question (see Methods for details).

The subplots correspond to the following open-ended questions:

i, Q19 – On a scale of 1–10, how helpful were HopeBot’s recommendations? What influenced your rating?

j, Q20 – On a scale of 1–10, how clear was HopeBot’s voice output? Did its tone match your expectations? Were you willing to listen to the entire response? Please provide the reasoning behind your rating.

k, Q22 – In your experience using HopeBot, in what aspects do you believe its mental health support services approach the level of professional mental health practitioners? Conversely, in which areas do you find it still difficult to serve as a substitute? Please elaborate on your perspective.

l, Q23 – What were HopeBot’s strengths and weaknesses, and how could it better support mental health and depression screening?

m, Q24 – Would you feel comfortable using HopeBot in the future or suggesting it to someone else? What factors influence your decision?

n, Q25 – How do you see AI and technology shaping the future of mental health screening and support?

This figure supplements the qualitative findings by illustrating the relative distribution and prominence of user-reported experiences, preferences, and suggestions across several key domains of chatbot functionality, perceived professionalism, strengths and weaknesses, future willingness, and broader perspectives on AI in mental health.
