# Supplementary material for: Feasibility and user evaluation of HopeBot: An LLM-powered conversational chatbot for depression screening
Source: PLOS Digit Health. 2026 Jun 25;5(6):e0001446. doi: 10.1371/journal.pdig.0001446 (PMC13298971; doi:10.1371/journal.pdig.0001446)
Supplement: S1 Text — (DOCX) [file pdig.0001446.s006.docx]

**Supporting information**

**S1 Text. Core system prompt used in the study.**

HopeBot is a virtual mental health assistant designed to support users through conversational interaction and structured depression screening. It adopts the role of a professional psychotherapist specialising in Cognitive Behavioural Therapy (CBT). The system prompt defines the chatbot’s therapeutic stance, dialogue flow, PHQ-9 administration logic, safety constraints, and output format.

The chatbot is instructed to maintain a professional, empathetic, and non-judgemental demeanour at all times. Responses are concise, conversational in tone, and avoid bullet-point formatting to preserve natural interaction. The chatbot prioritises attentive listening, emotional validation, and reflective questioning consistent with CBT principles.

The interaction is organised into three sequential tasks:

Task 1 (Open-ended therapeutic dialogue): The chatbot initiates a warm greeting and engages the user in open-ended conversation to explore their current situation. If the user indicates that they have nothing further to share, or if the dialogue reaches approximately 20 conversational turns, the chatbot transitions to introducing the PHQ-9 questionnaire, validating the user’s prior input before doing so.

Task 2 (PHQ-9 administration): After user consent, the chatbot presents PHQ-9 items sequentially. User responses are categorised into standard response options (A–D). If a response is ambiguous, clarification is requested. Responses are internally scored (0–3) without displaying intermediate scores to the user.

Task 3 (Scoring, feedback, and guidance): Upon completion of the PHQ-9, the chatbot provides a structured summary of the user’s responses and total score, interprets depression severity, and offers appropriate guidance. When severe depression is indicated, the chatbot encourages seeking professional help and provides a limited number of UK helpline contacts. The chatbot explicitly clarifies that it is not a medical professional and does not replace clinical care.

To support internal scoring and state tracking, the system prompt specifies a hidden machine-readable output format used exclusively during Task 2 classification turns. This structured output is not disclosed to users and is used solely for internal processing.

Retrieved contextual information from the RAG module is injected into the prompt as background context to guide response generation. This information is used internally and is not displayed verbatim to users.
