# Supplementary material for: Feasibility and user evaluation of HopeBot: An LLM-powered conversational chatbot for depression screening
Source: PLOS Digit Health. 2026 Jun 25;5(6):e0001446. doi: 10.1371/journal.pdig.0001446 (PMC13298971; doi:10.1371/journal.pdig.0001446)
Supplement: S2 Text — (DOCX) [file pdig.0001446.s007.docx]

**Supporting information**

**S2 Text. Ethics, safety, and privacy.**

HopeBot was explicitly framed as a supportive screening tool rather than a diagnostic or emergency service and was designed to communicate in a non-judgmental and empathetic manner.

The system used predefined keyword triggers to detect psychological crises, such as references to self-harm or acute emotional distress. When activated, it interrupted the session, delivered a pre-scripted supportive message, and redirected users to appropriate mental health services. A curated list of country-specific resources was integrated: for UK users, this included Samaritans (116 123) and Shout (text 85258); for users in China, it included major psychiatric institutions such as Peking University Sixth Hospital and Shanghai Mental Health Centre. All safety protocols and referral procedures were approved by the UCL Research Ethics Committee under a high-risk ethics application.

To ensure privacy and data security, all interactions were anonymised and stored in compliance with the General Data Protection Regulation. No personally identifiable data was collected, and OpenAI’s API settings were configured to prevent data retention. Data access was restricted to authorised research personnel and subject to participant consent.
