# Supplementary material for: Feasibility and user evaluation of HopeBot: An LLM-powered conversational chatbot for depression screening
Source: PLOS Digit Health. 2026 Jun 25;5(6):e0001446. doi: 10.1371/journal.pdig.0001446 (PMC13298971; doi:10.1371/journal.pdig.0001446)
Supplement: S3 Text — (DOCX) [file pdig.0001446.s008.docx]

**Supporting information**

**S3 Text. 25-item questionnaire.**

1. What is your age?
2. With which gender do you identify most?
3. What is your ethnic background?
4. What is your level of education?
5. What is your employment status?
6. Can you tell me a little bit about your background, including any experience you've had with mental health support or chatbots?
7. How familiar are you with large language models, such as ChatGPT, Gemini, Bing or similar AI technologies?
8. During your interaction with HopeBot, was there anything that stood out to you—either positively or negatively? Could you describe a specific moment or feature that made an impression on you and explain why?
9. Were there moments during your conversations with HopeBot when you felt truly understood and heard? Were there times when its responses felt a bit off or robotic? How did these experiences impact you?
10. What is your Patient Health Questionnaire-9 (PHQ-9) self-reported result?
11. What is your PHQ-9 result, as assessed with the assistance of HopeBot?
12. Is there a difference between the results of your PHQ-9 self-test and the scores completed with the assistance of HopeBot? If so, what do you think might have caused this discrepancy? Which results do you find more trustworthy, and why?
13. How does using HopeBot to complete the PHQ-9 compare to completing it alone at home? Which did you prefer, and why?
14. Did HopeBot introduce the PHQ-9 in a natural way? Were there any moments when its introduction felt disruptive or interrupted your conversation? Please share your thoughts.
15. Were the PHQ-9 questions easy to understand and answer? If anything was unclear, did you try asking HopeBot for help? And while you were responding, did HopeBot offer any useful support?
16. Did your conversation with HopeBot feel natural? Were there any points where the flow felt forced or responses seemed robotic?
17. On a scale of 1-10, how well did HopeBot handle sensitive topics related to depression? What influenced your rating?
18. On a scale of 1-10, how comfortable did you feel expressing your feelings without judgment? What influenced your rating?
19. On a scale of 1-10, how helpful were HopeBot’s recommendations? What influenced your rating?
20. On a scale of 1 to 10, how clear was HopeBot’s voice output? Did its tone match your expectations? Were you willing to listen to the entire response? Please provide the reasoning behind your rating.
21. Do you prefer using voice or text when chatting with HopeBot? Why? Which one feels more natural, like talking to a real person, and which is more convenient for you?
22. In your experience using HopeBot, in what aspects do you believe its mental health support services approach the level of professional mental health practitioners? Conversely, in which areas do you find it still difficult to serve as a substitute? Please elaborate on your perspective.
23. What were HopeBot’s strengths and weaknesses, and how could it better support mental health and depression screening?
24. Would you feel comfortable using HopeBot in the future or suggesting it to someone else? What factors influence your decision?
25. How do you see AI and technology shaping the future of mental health screening and support?
