# Supplementary material for: Feasibility and user evaluation of HopeBot: An LLM-powered conversational chatbot for depression screening
Source: PLOS Digit Health. 2026 Jun 25;5(6):e0001446. doi: 10.1371/journal.pdig.0001446 (PMC13298971; doi:10.1371/journal.pdig.0001446)
Supplement: S4 Text — (DOCX) [file pdig.0001446.s009.docx]

**Supporting information**

**S4** **Text. Codebook.**

This supplementary material presents the complete qualitative codebook developed for thematic analysis of user experiences with HopeBot. The qualitative analysis covered a total of 18 core qualitative questions (Q7–Q9, Q12–Q14, Q16-Q20, Q22–Q25) and provided coding guidance for additional optional questions.

Q7 Familiarity with large language models

Theme 1 Technical Familiarity — Codes: NO_EXPERIENCE, HEARD_ONLY, OCCASIONAL_USER, REGULAR_USER, TECHNICAL_EXPERT

Q8 Memorable moments during HopeBot use
Theme 1 User Experience & Usability — Codes: Alt-tool preference; Desire for more interaction; Dislike text-reading; Easy to use; General positive impression; No special impression; Novel & unique experience; Skill-dependent usability
Theme 2 Human-likeness & Realism — Codes: Feeling cared for; Genuine & meaningful interaction; Human-like interaction
Theme 3 Voice Quality & Audio — Codes: Desire for voice-output control; Positive voice experience; Unnatural voice; Voice annoyance / unnecessary output; Voice overlap / disruption; Voice timing issue

Theme 4 Interaction Flow & Responsiveness — Codes: Direct questions; Disrupted conversation; Lack depth / analysis; Lack personalisation; Quick & instant response; Responsive interaction; Slow response / delay; Smooth & engaging conversation
Theme 5 Emotional Support & Empathy — Codes: Affirmation / validating feelings; Emotional support; Encouragement; Guidance toward positive reflection / action; Response to emotional distress
Theme 6 Trust & Privacy — Codes: Disclosure control; Feeling analysed; Privacy concern
Theme 7 Modality Preference — Codes: Typing preference; Voice-to-text preference
Theme 8 Helpfulness & Content — Codes: Adequate amount of advice; Generic / inadequate response; Specific & personalised advice; Targeted health & sleep advice; Timely & relevant suggestions
Theme 9 Technical & Accuracy — Codes: Accurate voice recognition; System functionality issues; Voice recognition problems

Q9 Feeling understood versus robotic
Theme 1 Feeling Understood & Heard —Codes: Affirmation & validation; Contextual response; Deep empathy; Emotional support; Empathetic questioning; Feeling cared for/understood; Genuine & meaningful interaction; Human-like interaction; Encouraged sharing/increased willingness to share

Theme 2 Feeling “Off” or Robotic — Codes: Generic/ inadequate/ formulaic/ repetitive replies; Lack of personalisation; Lack of depth/ analysis; No emotional connection; Stiff or monotonous tone; Mechanical/ unnatural voice delivery; Desire for greater voice-output control

Theme 3 Emotional/ Psychological Impact — Codes: Positive emotional impact & stress relief; Neutral/ mixed emotional impact; Negative emotional impact; Emotional encouragement; Guidance toward positive reflection/ action

Theme 4 Interaction & Trust Dynamics — Codes: Quick & instant response; Slow response/ delay; Smooth & engaging conversation; Increased sharing/ openness; Reduced engagement/ willingness to engage; Trust shift/ no attachment

Theme 5 Perceived Quality & Expectations — Codes: Overall satisfaction/ general positive impression; Specific & personalised advice; Timely & relevant suggestions; Targeted health & sleep advice; Need for more human-like features; Tool comparison/ perception of professionalism; Clinical perception; Requests for specific improvements

Q12 Difference between PHQ-9 scores
Theme 1 Reasons for Difference — Codes: R-DEEPER, S-TIME, S-VOICE_ERR, S-TECH_LIMIT, R-EMO_SUP, R-GUIDE, S-PRIVACY, S-MOOD, S-INTUIT

Q13 HopeBot versus self-assessment
Theme 1 Process Efficiency — Codes: P-FAST, P-SLOW, P-EASY
Theme 2 Interaction Experience — Codes: I-ENGAGE, I-HUMAN, I-NONJUDGE
Theme 3 Cognitive & Result Quality — Codes: C-GUIDE, C-FEEDBK, C-CONTEXT, C-OBJECT, C-INTUIT
Theme 4 Risk & Concerns — Codes: R-PRIVACY, R-TECH, R-FUTURE

Q14 Introduction of PHQ-9 within conversation
Theme 1 Introduction Flow — Codes: INTRO-SMOOTH, INTRO-RUSHED, INTRO-PROMPTED, INTRO-MISSED
Theme 2 Conversation Disruption — Codes: DISR-NONE, DISR-CUT, DISR-PACING, DISR-UI
Theme 3 Tone/Naturalness — Codes: TONE-HUMAN, TONE-MECH

Q16 Naturalness of conversation
Theme 1 Tone — Codes: TONE-HUMAN, TONE-ROBOT, TONE-MIX
Theme 2 Conversation Flow — Codes: FLOW-SMOOTH, FLOW-DISJOINT, FLOW-REPEAT, FLOW-PACING
Theme 3 Understanding & Appropriateness — Codes: UNDERSTAND-GOOD, UNDERSTAND-MISS
Theme 4 Voice & Interface — Codes: VOICE-NAT, VOICE-ROBOT, UI-ISSUE

Q17 Handling sensitive depression topics
Theme 1 Positive Factors — Codes: A-EMPATH, A-VALIDATE, A-ADVICE, A-SAFE, A-FLOW
Theme 2 Limitations — Codes: B-ROBOT, B-SHALLOW, B-REPEAT, B-SLOW, B-RUSH, B-SELF

Q18 Comfort expressing feelings
Theme 1 Comfort Factors — Codes: CF-ANON, CF-NEUT, CF-EMPA, CF-AFFIRM, CF-SAFE, CF-GUIDE
Theme 2 Discomfort Factors — Codes: DF-PRIV, DF-ROBOT, DF-SHAL, DF-MISINT, DF-RUSH, DF-SELF, DF-NOHELP

Q19 Helpfulness of recommendations
Theme 1 Helpful Factors — Codes: HP-ACTION, HP-RESOURCE, HP-PERSONAL, HP-VALIDATE, HP-SUMMARY, HP-ACCESS, HP-SOOTHE, HP-ACCURATE
Theme 2 Unhelpful Factors — Codes: HN-GENERIC, HN-NONE, HN-REPEAT, HN-SHALLOW, HN-UNFIT, HN-LENGTH, HN-SLOW, HN-SELF

Q20 Voice clarity and tone
Theme 1 Positive Contributors — Codes: VC-CLEAR, VC-CALM, VC-MATCH, VC-HUMAN, VC-ENGAGE, VC-ACCESS
Theme 2 Negative Contributors — Codes: VN-ROBOT, VN-SLOW, VN-LONG, VN-SEQ, VN-AUTO, VN-MISMATCH, VN-CLARITY, VN-LANG, VN-TEXTPREF, VN-TIME

Q22 HopeBot compared with professional practitioners
Theme 1 Professional-like Areas — Codes: PRO-KNOW, PRO-EMPATH, PRO-STRUCT, PRO-REFLECT, PRO-ACCESS, PRO-RESOURCE, PRO-CALM
Theme 2 Limitations — Codes: LIM-HUMAN, LIM-NONVERB, LIM-PERSONAL, LIM-DEPTH, LIM-ADAPT, LIM-EMOTION, LIM-TECH, LIM-TRUST, LIM-SUB

Q23 Strengths, weaknesses, and improvements
Theme 1 Strengths — Codes: PRO-Access, PRO-Privacy, PRO-NonJudg, PRO-Struct, PRO-Know, PRO-Empath, PRO-Speed, PRO-Voice, PRO-Screen, PRO-Guide
Theme 2 Limitations — Codes: LIM-NonVerb, LIM-Depth, LIM-Personal, LIM-Empath, LIM-Robot, LIM-Slow, LIM-Tech, LIM-Security, LIM-Scope, LIM-Trust, LIM-Voice
Theme 3 Improvements — Codes: IMP-Speed, IMP-UX, IMP-Warmth, IMP-Personal, IMP-Depth, IMP-NonVerb, IMP-Privacy, IMP-Resources, IMP-VoiceOpt, IMP-Engage

Q24 Future use and recommendation
Theme 1 Willingness — Codes: PRO-USE, PRO-REC, PRO-DUAL, COND-TRY, LIM-NO, LIM-PREFALT
Theme 2 Reasons — Codes: PRO-Free, PRO-Access, PRO-Quick, PRO-Easy, LIM-Slow, PRO-Privacy, LIM-Privacy, PRO-Help, PRO-Comfort, LIM-Depth, LIM-Scope, PRO-Warm, LIM-Robot, LIM-UX, IMP-Feature, IMP-Emotion, IMP-Accuracy

Q25 Future of AI in mental health
Theme 1 Benefits — Codes: ADV-Access, ADV-Speed, ADV-EarlyDetect, ADV-Scale, ADV-Anonym, ADV-Cost, ADV-Companion, ADV-Personal, ADV-Tool
Theme 2 Risks — Codes: LIM-Accuracy, LIM-Depth, LIM-Privacy, LIM-Ethics, LIM-Replace, LIM-Sensitive, LIM-UX, LIM-Regulate, LIM-Context, LIM-Alt
Theme 3 Improvements — Codes: IMP-Improve, IMP-Interface, IMP-PrivacySafe, IMP-Supervise, IMP-Personalise, IMP-Crisis
Theme 4 Meta-stances — Codes: BAL-Mixed, UNC-Unknown
