# Supplementary material for: Feasibility and user evaluation of HopeBot: An LLM-powered conversational chatbot for depression screening
Source: PLOS Digit Health. 2026 Jun 25;5(6):e0001446. doi: 10.1371/journal.pdig.0001446 (PMC13298971; doi:10.1371/journal.pdig.0001446)
Supplement: Attachment — Submitted filename: Comment to authors.docx [file pdig.0001446.s010.docx]

**General Assessment**

This manuscript presents a timely and relevant feasibility study evaluating *HopeBot*, an LLM-powered conversational chatbot designed for depression screening. The integration of large language models into mental health screening tools is an important and emerging area within digital health, and the authors make a meaningful contribution by focusing on usability, user acceptance, and feasibility rather than diagnostic replacement.

Overall, the study is clearly motivated, ethically grounded, and aligned with the scope of *PLOS Digital Health*. However, several methodological and reporting aspects require clarification or strengthening to support the robustness and interpretability of the findings.

### Minor Comments

- Minor grammatical and typographical errors should be corrected during revision.
- Some technical terms related to large language models may benefit from brief definitions for a multidisciplinary readership.
- Figures and tables are generally clear but could include more descriptive captions to be interpretable independently.
- The analysis is largely descriptive, which is acceptable for feasibility work; however, reporting effect sizes, confidence intervals, or basic inferential statistics where applicable would improve interpretability
- The authors should clarify how missing data were handled, if applicable.
- The study relies heavily on self-reported user feedback. Consider discussing whether any validated depression screening instruments (e.g., PHQ-9) were used for comparison or calibration, even if only exploratorily.
- If no clinical benchmark was used, this limitation should be more explicitly stated.
