# Supplementary material for: Feasibility and user evaluation of HopeBot: An LLM-powered conversational chatbot for depression screening
Source: PLOS Digit Health. 2026 Jun 25;5(6):e0001446. doi: 10.1371/journal.pdig.0001446 (PMC13298971; doi:10.1371/journal.pdig.0001446)
Supplement: Attachment — Submitted filename: Response to Reviewers.docx [file pdig.0001446.s012.docx]

**Answers to the Reviewers**

Please note that we included the corresponding context changes in the manuscript for each question and highlighted them in red. Additionally, we have shown our revised sections in the format Revised Manuscript:" (revised part) ".

**Reviewer 1:**

- Voice input processing: You mention "automatic speech recognition module" but don't specify which ASR system was used. Was this OpenAI's Whisper? Please clarify.

**Author response:** Thank you for this helpful comment. We have clarified the speech recognition component in the system description by explicitly stating that voice input was processed using OpenAI’s Whisper automatic speech recognition model (Methods, System Design section).

Revised Manuscript: “Voice input was processed using OpenAI’s Whisper [37] automatic speech recognition model, and system responses were synthesised into audio using the OpenAI TTS-1 text-to-speech system [38].”

- RAG retrieval parameters: More technical detail would enhance reproducibility, such as Top-k value used for retrieval
  similarity threshold, reranking strategies, how retrieved passages were integrated into prompts.

**Author response:** Thank you for this constructive suggestion. We have expanded the Methods section to include additional implementation details of the RAG pipeline to enhance reproducibility. Specifically, we now report that retrieval is performed across three Chroma vector stores using the user’s most recent utterance as the query, with a top-k value of 2 per store (up to six passages per turn). We clarify that no additional similarity thresholding or reranking was applied, and we describe how retrieved passages are concatenated and injected into the system prompt as contextual grounding prior to generation.

Revised Manuscript: “At each conversational turn, the user’s most recent utterance was used as the retrieval query. Retrieval was performed across three Chroma vector stores corresponding to the curated CBT corpus, public emotional-support datasets, and helpline resources. For each store, the top-k most relevant passages were retrieved (k = 2 per store; up to six passages per turn). No additional similarity thresholding or reranking was applied.

The retrieved passages were concatenated in newline-separated form and injected into the system prompt as contextual grounding, alongside the full conversational history.”

- Prompt engineering: While you mention "structured prompt engineering," the actual prompts are not provided. Consider including core system prompts in supplementary materials and documenting prompt iterations during the expert review phase. This is also critical for reproducibility.

**Author response:** Thank you for this helpful suggestion. We have now included the final core system prompt governing HopeBot’s behaviour as S3 Text to improve transparency and reproducibility. The Methods section has been updated to explicitly reference this appendix and to clarify that the system prompt was iteratively refined during expert review prior to deployment. Former supplementary materials have been renumbered accordingly.

Revised Manuscript: “Response generation was governed by a structured, task-oriented system prompt that explicitly controlled dialogue flow, PHQ-9 item administration, response classification, scoring logic, and safety constraints. The augmented prompt was then passed to GPT-4o to generate responses. The system prompt was iteratively refined during expert review and fixed prior to deployment; the final version is provided in S3 Text.”

- The 86% inter-coder agreement with independent second coder is excellent. Consider providing more detail on disagreement resolution process

**Author response:** Thank you for this positive and constructive comment. We have revised the Methods section to provide additional detail on how disagreements were resolved. Specifically, we now clarify that discrepancies in code assignment or theme mapping were addressed through iterative discussion between coders, involving joint review of the original responses, clarification of code definitions within the codebook, and refinement of theme boundaries until consensus was reached.

Revised Manuscript: “Discrepancies in code assignment or theme mapping were resolved through iterative discussion between coders, involving joint review of the original responses, clarification of code definitions within the codebook, and refinement of theme boundaries until consensus was reached.”

- It may be beneficial to give a brief discussion of resource requirements for deployment and average interaction time e.g. 25 mins for HopeBot vs. "X" mins for self-report. Offer suggestions in settings where the additional time investment might be justified if any.

**Author response:** Thank you for this helpful suggestion. We have added a brief discussion in the Discussion section addressing practical deployment considerations, including a comparison of average interaction time between HopeBot and self administered PHQ-9, as well as resource requirements for cloud based deployment. We also outline settings in which the additional time investment associated with conversational screening may be justified, such as contexts prioritising engagement, clarification, or psychological support.

Revised Manuscript: “From a practical deployment perspective, the PHQ-9 remains highly efficient in self-administered form, typically requiring approximately 2–3 minutes to complete [66]. By contrast, internal pilot testing of HopeBot indicated a substantially longer interaction time. Across ten internal test sessions, a complete HopeBot interaction, including open-ended dialogue and PHQ-9 administration, lasted a mean of 25 minutes and 23 seconds, with duration varying according to the extent of user self-disclosure and conversational depth.

This increased time investment reflects the system’s design emphasis on open-ended dialogue, clarification, and emotional support rather than rapid item completion. While less suitable for high-throughput screening, such an approach may be justified in contexts where engagement, comprehension, or psychological reassurance are prioritised, including remote or underserved settings, populations with low mental health literacy, or individuals reluctant to complete standard questionnaires. In these scenarios, conversational screening may complement brief self-report instruments rather than replace them.

In terms of deployment requirements, HopeBot does not require specialised hardware on the user side, as computation is handled via cloud-based language model inference and lightweight vector retrieval. Operational costs are therefore primarily associated with API usage (including language model inference and optional speech transcription and synthesis), alongside standard hosting and data management. These costs can be moderated through design choices such as default text-based interaction, constrained retrieval context, and session-length limits, depending on deployment setting and scale.”

- GPT-4o model version: Large language models are frequently updated. Please specify the exact API version or model checkpoint used, the evaluation dates relative to model updates and whether response consistency was tested across the study period.

**Author response:** Thank you for this important comment. We have revised the Methods section to clarify the GPT-4o model configuration, study time window, and version control strategy. Specifically, all interactions used the same GPT-4o API endpoint with a fixed system prompt during a defined study period, and response consistency was monitored during internal testing to ensure stable task flow and scoring logic. These details have now been explicitly documented in the revised manuscript.

Revised Manuscript: “All interactions were generated using the OpenAI GPT-4o model accessed via the OpenAI API (model identifier: “gpt-4o”) [39]. Data collection and system evaluation were conducted within a fixed study window (March–April 2025), during which the same model endpoint and prompt configuration were used throughout. To minimise the impact of model updates, the system prompt was fixed prior to data collection, and no adaptive prompt or model changes were introduced during the study period. While response stochasticity is inherent to large language models, system behaviour was monitored during internal testing through repeated end-to-end trial runs and manual review of conversation logs to verify consistent task sequencing, PHQ-9 item coverage, and scoring logic. No substantive deviations in task flow or score computation were observed during the study period.”

- The manuscript notes that "Mandarin outputs were produced from Chinese prompts" but provides limited analysis of cross-linguistic performance. Consider separate concordance statistics for English vs. Mandarin interactions, alongside discussion of any language-specific challenges observed or whether voice synthesis quality differed between languages (you mention this was an issue)

**Author response:** Thank you for this insightful comment. In the present study, the language of interaction was fully aligned with the country of recruitment: participants recruited in the UK completed all interactions in English, whereas participants recruited in China completed all interactions in Mandarin. Although the study was not designed as a formal cross-linguistic validation, robustness across language contexts was explicitly examined through subgroup analyses already reported in the main Results section.

Specifically, subgroup analyses by recruitment country (reported in the Results section under PHQ-9 Score Concordance) demonstrated high concordance between HopeBot-assisted and self-administered PHQ-9 scores in both the UK and China cohorts. While UK participants showed slightly higher concordance metrics and a smaller median absolute deviation than Chinese participants, the observed differences were small in magnitude and unlikely to be clinically meaningful, supporting stable screening performance across language and cultural contexts. These findings are reported in the Results section under subgroup robustness analyses.

Revised Manuscript: “To test robustness across participant groups, subgroup analyses were conducted by baseline severity and recruitment country. Concordance remained high in both mild (PHQ-9 < 10: ρ = 0.858, ICC = 0.845) and moderate-to-severe participants (≥10: ρ = 0.711, ICC = 0.785), with no significant difference in absolute score deviation (U = 1579.5, p = 0.219, r = 0.10), indicating a negligible effect size. Across countries, UK participants showed a slightly smaller median deviation (0 vs 1 point; IQR 1.00 vs 2.00) and higher concordance (ρ = 0.938 vs 0.892; ICC = 0.942 vs 0.883). A modest but statistically significant Mann–Whitney difference was observed (U = 2645.0, p = 0.023, r = 0.19). However, the effect size was small, suggesting that the observed difference is unlikely to be clinically meaningful. Overall, these findings support stable performance across cultural and symptom-severity strata.”

In addition, user-rated experience measures (Q17–Q20) did not differ significantly by country or ethnicity (Table 2), suggesting that perceived usability, trust, voice clarity, and recommendation helpfulness were not systematically influenced by language background.

With respect to language-specific challenges, these were primarily observed at the level of voice output rather than score administration. As discussed in the Technical, Ethical, and Linguistic Constraints section, Mandarin speech synthesis relied on a general-purpose text-to-speech engine optimised for English prosody, which occasionally introduced prosodic inconsistencies and reduced perceived naturalness. Importantly, these issues were qualitative in nature and did not affect PHQ-9 item coverage, scoring logic, or concordance outcomes.

Taken together, these results indicate that HopeBot’s scoring fidelity and overall user experience were robust across English- and Mandarin-speaking participants within this cohort. We agree that future work should include dedicated cross-linguistic validation with language-stratified concordance metrics and culturally optimised speech synthesis models, and we have clarified this scope explicitly in the Study Limitations section of the revised manuscript.

Revised Manuscript: “Fourth, although participants were recruited from both the UK and China, the study was not designed or powered to formally evaluate cultural, linguistic, or cross-national differences in interaction patterns or user perceptions. Accordingly, subgroup analyses were exploratory and should not be interpreted as evidence of cross-cultural equivalence.”

**Reviewer 2:**

- Minor grammatical and typographical errors should be corrected during revision.

**Author response:** We thank the reviewer for this comment. Minor grammatical, typographical, and spelling inconsistencies have been reviewed and corrected throughout the manuscript, with spelling standardised to British English for consistency.

- Some technical terms related to large language models may benefit from brief definitions for a multidisciplinary readership.

**Author response:** We thank the reviewer for this helpful suggestion. To improve accessibility for a multidisciplinary readership, we have added brief, functional definitions at the first occurrence of selected large language model–related technical terms (e.g., large language models, retrieval-augmented generation, and intraclass correlation coefficient), while avoiding unnecessary implementation-level detail. Below is an example of these explanations:

Revised Manuscript: “Conversational agents powered by large language models (LLMs)—neural network–based systems designed to generate and interpret natural language—have emerged as a promising means of addressing limitations in traditional mental health screening [18].”

- Figures and tables are generally clear but could include more descriptive captions to be interpretable independently.

**Author response:**  We thank the reviewer for this helpful suggestion. Figure and table captions have been revised to improve standalone interpretability for a multidisciplinary readership. Specifically, captions were expanded to clarify key visual elements (e.g., reference lines in agreement plots), summarise the purpose of each panel, and provide concise methodological context where appropriate (e.g., statistical tests used in tables), while avoiding unnecessary implementation-level detail. These revisions allow the figures and tables to be understood independently of the main text.

- The analysis is largely descriptive, which is acceptable for feasibility work; however, reporting effect sizes, confidence intervals, or basic inferential statistics where applicable would improve interpretability

**Author response:** We thank the reviewer for this constructive suggestion. In response, we have strengthened the statistical reporting in several specific sections of the manuscript to improve interpretability beyond descriptive summaries.

**1. Subgroup concordance analyses (Results section).**

We clarified the inferential testing framework and now explicitly report effect size measures alongside test statistics and p-values. In addition to Spearman’s ρ and ICC for concordance, Mann–Whitney U comparisons are accompanied by effect size r, allowing clearer interpretation of the magnitude and practical relevance of subgroup differences.

**2. Univariable demographic analyses (Table 2 and corresponding Results text).**

For all inferential tests examining associations between demographic characteristics and user ratings (Q17–Q20), appropriate effect size measures have now been added. Specifically, Cohen’s d is reported for independent-samples t tests (binary variables), and η² is reported for one-way ANOVA (multilevel variables). Table 2 has been updated to include these additional columns, and the caption has been revised to define the reported effect size metrics. Statistically significant p values are indicated in bold for clarity.

**3. Methods section (Data Analysis Method subsection).**

The Methods have been revised to explicitly state the statistical tests used, the corresponding effect size measures, and the criteria for interpretation. This ensures alignment between the analytical framework and the reported results.

Across comparisons, most demographic effects were small in magnitude (η² ≤ 0.062; |d| ≤ 0.30), indicating limited demographic influence on perceived usability and emotional tone. The only moderate effects observed were for employment status and prior mental health treatment experience in relation to perceived helpfulness (Q19), which are now explicitly contextualised in the Results section.

We hope these revisions adequately address the reviewer’s concern and improve the interpretability and transparency of the statistical analyses

- The authors should clarify how missing data were handled, if applicable.

**Author response:** We thank the reviewer for this comment. Missing data were handled through predefined quality screening criteria rather than post hoc statistical handling. Submissions that were substantially incomplete (<80% overall questionnaire completion) were excluded prior to analysis. In the final analytic sample, all PHQ-9 items were completed in both the self-administered and HopeBot-assisted assessments. For user evaluation items, non-numeric responses (e.g., “I don’t know”) were retained in the dataset but excluded from item-level quantitative analyses, which were conducted using available numeric responses only. These clarifications have been added to the Methods section.

Revised Manuscript: “In the final analytic sample, all PHQ-9 items were completed, and no item-level missing data were present in either the self-administered or HopeBot-assisted assessments. For Likert-scale user evaluation items (e.g., Q17–Q20), non-numeric responses such as “I don’t know” were retained in the dataset but excluded from item-level quantitative analyses; summary statistics and tests were computed using available numeric responses only, without imputation.”

- The study relies heavily on self-reported user feedback. Consider discussing whether any validated depression screening instruments (e.g., PHQ-9) were used for comparison or calibration, even if only exploratorily.

**Author response:** We thank the reviewer for this thoughtful suggestion. A validated depression screening instrument (PHQ-9) was incorporated throughout the study not only as the assessment content, but also as a reference framework for evaluating format-level consistency in administration. Specifically, self-administered PHQ-9 scores were used as a pragmatic comparator to assess the fidelity of HopeBot-assisted administration, rather than to establish diagnostic validity.

To clarify this methodological positioning, we have strengthened the description in both the Methods and Discussion sections. For example, we now explicitly state:

Revised Manuscript: “Importantly, the PHQ-9 served as a validated screening framework for evaluating format-level consistency in administration rather than as a diagnostic gold standard, which remains clinician-administered interviews.”

In addition, we have expanded the Discussion to emphasise that user evaluation items capture subjective interaction experience and trust, while the PHQ-9 comparison provides a validated anchor for assessing technical and procedural fidelity. We further acknowledge that validation against clinician-led diagnostic interviews represents an important direction for future work. These revisions clarify the role of PHQ-9 as a calibration reference rather than a diagnostic benchmark.

- If no clinical benchmark was used, this limitation should be more explicitly stated.

**Author response:** We thank the reviewer for highlighting this point. We have now made this limitation more explicit in the manuscript. Specifically, we clarify that no clinician-administered diagnostic benchmark was used in the present study and that the validation framework focused on comparing HopeBot-assisted and self-administered PHQ-9 formats. For example, we now state in the Study Limitations section:

Revised Manuscript: “First, no clinician-administered diagnostic benchmark was used in the present study. The validation framework therefore compared HopeBot-assisted and self-administered PHQ-9 formats rather than clinician-led references; results reflect format-level concordance in administration rather than diagnostic validity.”

This revision explicitly delineates the scope of validation and the distinction between format-level fidelity and clinical diagnostic benchmarking.
