# Supplementary material for: Feasibility and user evaluation of HopeBot: An LLM-powered conversational chatbot for depression screening
Source: PLOS Digit Health. 2026 Jun 25;5(6):e0001446. doi: 10.1371/journal.pdig.0001446 (PMC13298971; doi:10.1371/journal.pdig.0001446)
Supplement: Peer review history

# Peer review: Decision Letter 0

**DOI:** 10.1371/journal.pdig.0001446.r001

**Authors:** Rachel Vanderkruik, Baki Kocaballi

9 Feb 2026

PDIG-D-25-00998

Feasibility and User Evaluation of HopeBot: An LLM-Powered Conversational Chatbot for Depression Screening

PLOS Digital Health

Dear Dr. Li,

Thank you for submitting your manuscript to PLOS Digital Health. After careful consideration, we feel that it has merit but does not fully meet PLOS Digital Health's publication criteria as it currently stands. Therefore, we invite you to submit a revised version of the manuscript that addresses the points raised during the review process.

Please submit your revised manuscript by Mar 11 2026 11:59PM. If you will need more time than this to complete your revisions, please reply to this message or contact the journal office at digitalhealth@plos.org. When you're ready to submit your revision, log on to https://www.editorialmanager.com/pdig/ and select the 'Submissions Needing Revision' folder to locate your manuscript file.

Please include the following items when submitting your revised manuscript:

* A letter that responds to each point raised by the editor and reviewer(s). You should upload this letter as a separate file labeled 'Response to Reviewers'. This file does not need to include responses to any formatting updates and technical items listed in the 'Journal Requirements' section below.

* A marked-up copy of your manuscript that highlights changes made to the original version. You should upload this as a separate file labeled 'Revised Manuscript with Track Changes'.

* An unmarked version of your revised paper without tracked changes. You should upload this as a separate file labeled 'Manuscript'.

If you would like to make changes to your financial disclosure, competing interests statement, or data availability statement, please make these updates within the submission form at the time of resubmission. Guidelines for resubmitting your figure files are available below the reviewer comments at the end of this letter.

We look forward to receiving your revised manuscript.

Kind regards,

Rachel Vanderkruik

Academic Editor

PLOS Digital Health

Baki Kocaballi

Section Editor

PLOS Digital Health

Leo Anthony Celi

Editor-in-Chief

PLOS Digital Health

orcid.org/0000-0001-6712-6626

**Journal Requirements:**

1. Please ensure that your Ethics Statement is available in its entirety at the beginning of your Methods section, under a subheading 'Ethics Statement'.

2. We ask that a manuscript source file is provided at Revision. Please upload your manuscript file as a .doc, .docx, .rtf or .tex.

3. Please upload separate figure files in .tif or .eps format. Also, remove the figures from your manuscript file but keep the legends.

For more information about figure files please see our guidelines:

https://journals.plos.org/digitalhealth/s/figures

https://journals.plos.org/digitalhealth/s/figures#loc-file-requirements

4. We notice that your Supporting Information are included in the manuscript file. Please remove them and upload them with the file type 'Supporting Information'. Please ensure that each Supporting Information file has a legend listed in the manuscript after the references list.

5. Some material included in your submission may be copyrighted. According to PLOS’s copyright policy, authors who use figures or other material (e.g., graphics, clipart, maps) from another author or copyright holder must demonstrate or obtain permission to publish this material under the Creative Commons Attribution 4.0 International (CC BY 4.0) License used by PLOS journals. Please closely review the details of PLOS’s copyright requirements here: PLOS Licenses and Copyright. If you need to request permissions from a copyright holder, you may use PLOS's Copyright Content Permission form.

Please respond directly to this email or email the journal office and provide any known details concerning your material's license terms and permissions required for reuse, even if you have not yet obtained copyright permissions or are unsure of your material's copyright compatibility.

Potential Copyright Issues:

a. Figures 1 and 2: Please confirm whether you drew the images / clip-art within the figure panels by hand. If you did not draw the images, please provide (a) a link to the source of the images or icons and their license / terms of use; or (b) written permission from the copyright holder to publish the images or icons under our CC-BY 4.0 license. Alternatively, you may replace the images with open source alternatives. See these open source resources you may use to replace images / clip-art:

- https://commons.wikimedia.org

- https://openclipart.org/

If the reviewer comments include a recommendation to cite specific previously published works, please review and evaluate these publications to determine whether they are relevant and should be cited. There is no requirement to cite these works unless the editor has indicated otherwise.

**Additional Editor Comments (if provided):**

[Note: HTML markup is below. Please do not edit.]

**Reviewers' Comments:**

Reviewer's Responses to Questions

**Comments to the Author**

1. Does this manuscript meet PLOS Digital Health’s publication criteria? Is the manuscript technically sound, and do the data support the conclusions? The manuscript must describe methodologically and ethically rigorous research with conclusions that are appropriately drawn based on the data presented.

Reviewer #1: Yes

Reviewer #2: Yes

**********

2. Has the statistical analysis been performed appropriately and rigorously?

Reviewer #1: Yes

Reviewer #2: Yes

**********

3. Have the authors made all data underlying the findings in their manuscript fully available (please refer to the Data Availability Statement at the start of the manuscript PDF file)?

The PLOS Data policy requires authors to make all data underlying the findings described in their manuscript fully available without restriction, with rare exception. The data should be provided as part of the manuscript or its supporting information, or deposited to a public repository. For example, in addition to summary statistics, the data points behind means, medians and variance measures should be available. If there are restrictions on publicly sharing data—e.g. participant privacy or use of data from a third party—those must be specified.

Reviewer #1: Yes

Reviewer #2: Yes

**********

4. Is the manuscript presented in an intelligible fashion and written in standard English?

PLOS Digital Health does not copyedit accepted manuscripts, so the language in submitted articles must be clear, correct, and unambiguous. Any typographical or grammatical errors should be corrected at revision, so please note any specific errors here.

Reviewer #1: Yes

Reviewer #2: Yes

**********

5. Review Comments to the Author

Please use the space provided to explain your answers to the questions above. You may also include additional comments for the author, including concerns about dual publication, research ethics, or publication ethics. (Please upload your review as an attachment if it exceeds 20,000 characters)

Reviewer #1: This manuscript represents thoughtful, rigorous research with important implications for scalable mental health screening. The authors demonstrate appropriate caution about AI limitations while documenting genuine potential of GenAI tools such as chatbots for supporting healthcare delivery.

The main strengths identified include:

1) Methodological Rigor: The study employs appropriate within-subject design with clear reporting following GRRAS guidelines. The mixed-methods approach combining quantitative concordance analysis with rich qualitative feedback is excellent.

2) Transparent Positioning: The authors are commendably transparent about positioning this as format-level validation rather than diagnostic validation. The explicit acknowledgment that self-administered PHQ-9 serves as a pragmatic comparator (not a gold standard) is crucial and well-articulated.

3) Technical Innovation: The RAG-based architecture grounding responses in validated CBT materials and helpline resources represents a thoughtful approach to reducing hallucination risks while maintaining conversational flexibility.

4) Comprehensive Evaluation: The 132-participant sample with detailed demographic stratification, combined with 25-item evaluation survey and thematic analysis following Braun & Clarke's framework, provides robust evidence.

5) Ethical Considerations: Strong attention to safety protocols, crisis handling, and appropriate boundaries of AI-mediated mental health support.

With the following suggested minor revisions, this will be a valuable contribution to the literature.

- Voice input processing: You mention "automatic speech recognition module" but don't specify which ASR system was used. Was this OpenAI's Whisper? Please clarify.

- RAG retrieval parameters: More technical detail would enhance reproducibility, such as Top-k value used for retrieval

similarity threshold, reranking strategies, how retrieved passages were integrated into prompts.

- Prompt engineering: While you mention "structured prompt engineering," the actual prompts are not provided. Consider including core system prompts in supplementary materials and documenting prompt iterations during the expert review phase. This is also critical for reproducibility.

- The 86% inter-coder agreement with independent second coder is excellent. Consider providing more detail on disagreement resolution process

- It may be beneficial to give a brief discussion of resource requirements for deployment and average interaction time e.g. 25 mins for HopeBot vs. "X" mins for self-report. Offer suggestions in settings where the additional time investment might be justified if any.

- GPT-4o model version: Large language models are frequently updated. Please specify the exact API version or model checkpoint used, the evaluation dates relative to model updates and whether response consistency was tested across the study period.

- The manuscript notes that "Mandarin outputs were produced from Chinese prompts" but provides limited analysis of cross-linguistic performance. Consider separate concordance statistics for English vs. Mandarin interactions, alongside

discussion of any language-specific challenges observed or whether voice synthesis quality differed between languages (you mention this was an issue)

Reviewer #2: his manuscript presents a timely and relevant feasibility study evaluating HopeBot, an LLM-powered conversational chatbot designed for depression screening. The integration of large language models into mental health screening tools is an important and emerging area within digital health, and the authors make a meaningful contribution by focusing on usability, user acceptance, and feasibility rather than diagnostic replacement.

Overall, the study is clearly motivated, ethically grounded, and aligned with the scope of PLOS Digital Health. However, several methodological and reporting aspects require clarification or strengthening to support the robustness and interpretability of the findings.

**********

6. PLOS authors have the option to publish the peer review history of their article (what does this mean?). If published, this will include your full peer review and any attached files.

**Do you want your identity to be public for this peer review?**   If you choose “no”, your identity will remain anonymous but your review may still be made public.

For information about this choice, including consent withdrawal, please see our Privacy Policy.

Reviewer #1: **Yes:**  Oluwaseun Ajao

Reviewer #2: No

**********

[NOTE: If reviewer comments were submitted as an attachment file, they will be attached to this email and accessible via the submission site. Please log into your account, locate the manuscript record, and check for the action link "View Attachments". If this link does not appear, there are no attachment files.]

**Figure resubmission:**

While revising your submission, we strongly recommend that you use PLOS’s NAAS tool (https://ngplosjournals.pagemajik.ai/artanalysis) to test your figure files. NAAS can convert your figure files to the TIFF file type and meet basic requirements (such as print size, resolution), or provide you with a report on issues that do not meet our requirements and that NAAS cannot fix.

After uploading your figures to PLOS’s NAAS tool - https://ngplosjournals.pagemajik.ai/artanalysis, NAAS will process the files provided and display the results in the "Uploaded Files" section of the page as the processing is complete. If the uploaded figures meet our requirements (or NAAS is able to fix the files to meet our requirements), the figure will be marked as "fixed" above. If NAAS is unable to fix the files, a red "failed" label will appear above. When NAAS has confirmed that the figure files meet our requirements, please download the file via the download option, and include these NAAS processed figure files when submitting your revised manuscript.

**Reproducibility:**

To enhance the reproducibility of your results, we recommend that authors of applicable studies deposit laboratory protocols in protocols.io, where a protocol can be assigned its own identifier (DOI) such that it can be cited independently in the future. Additionally, PLOS ONE offers an option to publish peer-reviewed clinical study protocols. Read more information on sharing protocols at https://plos.org/protocols?utm_medium=editorial-email&utm_source=authorletters&utm_campaign=protocols

Attachment Submitted filename: Comment to authors.docx

# Author comment: Author response to Decision Letter 1

**DOI:** 10.1371/journal.pdig.0001446.r002

27 Feb 2026

Attachment Submitted filename: Response to Reviewers.docx

# Peer review: Decision Letter 1

**DOI:** 10.1371/journal.pdig.0001446.r003

**Authors:** Rachel Vanderkruik, Baki Kocaballi

5 May 2026

Feasibility and User Evaluation of HopeBot: An LLM-Powered Conversational Chatbot for Depression Screening

PDIG-D-25-00998R1

Dear Dr. Li,

We are pleased to inform you that your manuscript 'Feasibility and User Evaluation of HopeBot: An LLM-Powered Conversational Chatbot for Depression Screening' has been provisionally accepted for publication in PLOS Digital Health.

Before your manuscript can be formally accepted you will need to complete some formatting changes, which you will receive in a follow-up email from a member of our team.

Please note that your manuscript will not be scheduled for publication until you have made the required changes, so a swift response is appreciated.

IMPORTANT: The editorial review process is now complete. PLOS will only permit corrections to spelling, formatting or significant scientific errors from this point onwards. Requests for major changes, or any which affect the scientific understanding of your work, will cause delays to the publication date of your manuscript.

If your institution or institutions have a press office, please notify them about your upcoming paper to help maximize its impact. If they'll be preparing press materials, please inform our press team as soon as possible -- no later than 48 hours after receiving the formal acceptance. Your manuscript will remain under strict press embargo until 2 pm Eastern Time on the date of publication. For more information, please contact digitalhealth@plos.org.

Thank you again for supporting Open Access publishing; we are looking forward to publishing your work in PLOS Digital Health.

Best regards,

Rachel Vanderkruik

Academic Editor

PLOS Digital Health

***********************************************************

**Additional Editor Comments (if provided):**

**Reviewer Comments (if any, and for reference):**

Reviewer's Responses to Questions

**Comments to the Author**

1. If the authors have adequately addressed your comments raised in a previous round of review and you feel that this manuscript is now acceptable for publication, you may indicate that here to bypass the “Comments to the Author” section, enter your conflict of interest statement in the “Confidential to Editor” section, and submit your "Accept" recommendation.

Reviewer #2: All comments have been addressed

**********

2. Does this manuscript meet PLOS Digital Health’s publication criteria? Is the manuscript technically sound, and do the data support the conclusions? The manuscript must describe methodologically and ethically rigorous research with conclusions that are appropriately drawn based on the data presented.

Reviewer #2: Yes

**********

3. Has the statistical analysis been performed appropriately and rigorously?

Reviewer #2: Yes

**********

4. Have the authors made all data underlying the findings in their manuscript fully available (please refer to the Data Availability Statement at the start of the manuscript PDF file)?

The PLOS Data policy requires authors to make all data underlying the findings described in their manuscript fully available without restriction, with rare exception. The data should be provided as part of the manuscript or its supporting information, or deposited to a public repository. For example, in addition to summary statistics, the data points behind means, medians and variance measures should be available. If there are restrictions on publicly sharing data—e.g. participant privacy or use of data from a third party—those must be specified.

Reviewer #2: Yes

**********

5. Is the manuscript presented in an intelligible fashion and written in standard English?

PLOS Digital Health does not copyedit accepted manuscripts, so the language in submitted articles must be clear, correct, and unambiguous. Any typographical or grammatical errors should be corrected at revision, so please note any specific errors here.

Reviewer #2: Yes

**********

6. Review Comments to the Author

Please use the space provided to explain your answers to the questions above. You may also include additional comments for the author, including concerns about dual publication, research ethics, or publication ethics. (Please upload your review as an attachment if it exceeds 20,000 characters)

Reviewer #2: The authors have adequately addressed previous concerns, significantly improving methodological transparency, statistical reporting, and clarity.

The study presents a well-designed feasibility and agreement analysis of an LLM-powered chatbot (HopeBot) for PHQ-9 administration. The within-subject design and use of multiple concordance metrics (ICC, Spearman correlation, and Bland–Altman analysis) provide strong evidence of reliable score replication.

Technical transparency is commendable, with clear descriptions of the system architecture, RAG pipeline, and prompt design, enhancing reproducibility. Statistical reporting is appropriate, with the inclusion of effect sizes and inferential tests improving interpretability.

The discussion clearly distinguishes between format-level concordance and diagnostic validity and appropriately acknowledges key limitations. Practical, ethical, and usability considerations are also well addressed.

Overall, this study makes a meaningful and timely contribution to digital mental health, demonstrating the feasibility and acceptability of conversational AI as a scalable screening tool aligned with the scope of PLOS Digital Health.

**********

7. PLOS authors have the option to publish the peer review history of their article (what does this mean?). If published, this will include your full peer review and any attached files.

**Do you want your identity to be public for this peer review?**   If you choose “no”, your identity will remain anonymous but your review may still be made public.

For information about this choice, including consent withdrawal, please see our Privacy Policy.

Reviewer #2: **Yes:**  Andrews Baidoo

**********
